# Thermal Stability of Aluminum Alloys

**DOI:** 10.3390/ma13153441

**Published:** 2020-08-04

**Authors:** Frank Czerwinski

**Affiliations:** CanmetMATERIALS, Natural Resources Canada, Hamilton, ON L8P 0A5, Canada; frank.czerwinski@canada.ca

**Keywords:** thermal stability, aluminum alloys, transition metals, rare earths, aerospace, automotive

## Abstract

Thermal stability, determining the material ability of retaining its properties at required temperatures over extended service time, is becoming the next frontier for aluminum alloys. Its improvement would substantially expand their range of structural applications, especially in automotive and aerospace industries. This report explains the fundamentals of thermal stability; definitions, the properties involved; and the deterioration indicators during thermal/thermomechanical exposures, including an impact of accidental fire, and testing techniques. For individual classes of alloys, efforts aimed at identifying factors stabilizing their microstructure at service temperatures are described. Particular attention is paid to attempts of increasing the current upper service limit of high-temperature grades. In addition to alloying aluminum with a variety of elements to create the thermally stable microstructure, in particular, transition and rare-earth metals, parallel efforts are explored through applying novel routes of alloy processing, such as rapid solidification, powder metallurgy and additive manufacturing, engineering alloys in a liquid state prior to casting, and post-casting treatments. The goal is to overcome the present barriers and to develop novel aluminum alloys with superior properties that are stable across the temperature and time space, required by modern designs.

## 1. Introduction

Thermal stability is the key design feature that determines a suitability of materials for specific applications and has a particular meaning for aluminum alloys. As documented throughout the decades, practically all aluminum alloys are thermally unstable with their properties being affected, to some extent, by service temperature and time. This includes grades essentially used at room temperatures, as is the case with aircraft components that may become warm due to exposure to sun, due to aerodynamic heating, or heat transferred from engines, which can deteriorate their properties over years of service [1]. The key engineering interest is, however, in the high temperature range and increasing the upper service limit of high-temperature grades [2].

At present, extending thermal stability to higher temperatures is the technology and knowledge barrier that prevents the substantial expansion of application scope of aluminum alloys, especially in automotive, marine, and aerospace transportation vehicles, designed for long-term service and strategically using aluminum for its lightweighting advantages. It is believed that the future aluminum alloys with improved high-temperature capabilities could compete, in selected applications, with more expensive titanium- and nickel-based grades. Therefore, along with recent refocusing on the strategic importance of aluminum alloys as lightweight structural materials for all forms of transportation vehicles, a substantial research interest is devoted to an improvement in their performance at high temperatures.

Although the thermal stability of aluminum alloys represents the major theme or at least a partial subject of a large number of research papers, differences in its understanding, critical property selection, and testing procedures make it difficult or impossible to combine individual results to draw a unified quantitative conclusion. The objective of this report is to review all elements of thermal stability from fundamentals to applications that refer to structural materials and aluminum alloys. Through identifying its detailed controlling factors, a better understanding of the relationship between mechanical, structural, and thermophysical properties that are critical for performance of alloys at increased temperatures will emerge. The outcome will help in optimizing the service conditions for existing aluminum alloys and development of novel alloys with superior thermal stability.

## 2. Defining Thermal Stability of Structural Materials and Aluminum

There is no universal definition or single criterion describing thermal stability of structural materials. While being typically seen as the “material ability of retaining its properties at required temperatures over extended service time”, in practice, more major parameters influencing thermal stability are involved including, in addition to (i) temperature and (ii) time, also (iii) load conditions and (iv) environmental conditions. Another definition as a “material resistance to permanent property changes caused by heat” is even less accurate as after cooling to room temperature, some portion of properties frequently recovers, whereas in a design, the properties maintained at the service temperature matter. Thermal stability is also defined as a material “property” characterizing changes after long-time exposure to elevated temperatures [3]. In this case, thermal stability is seen as an “intrinsic property” and, therefore, such an approach has further limitations. The related term “dimensional thermal stability” is also used that describes thermal expansion.

When assessing the thermal stability of a material, a future destination of this description is essential. If thermal stability is assessed for the purpose of comparing different alloys, e.g., during alloy development, the temperature and time are sufficient to characterize the alloy behavior. However, when a design input is required, during a material selection for a specific engineering application, detailed service conditions should be assessed. The design input will require an experimental measurement and/or computer simulation of material performance under conditions of its future application, including the load details and service environment nature.

**Figure 1:**
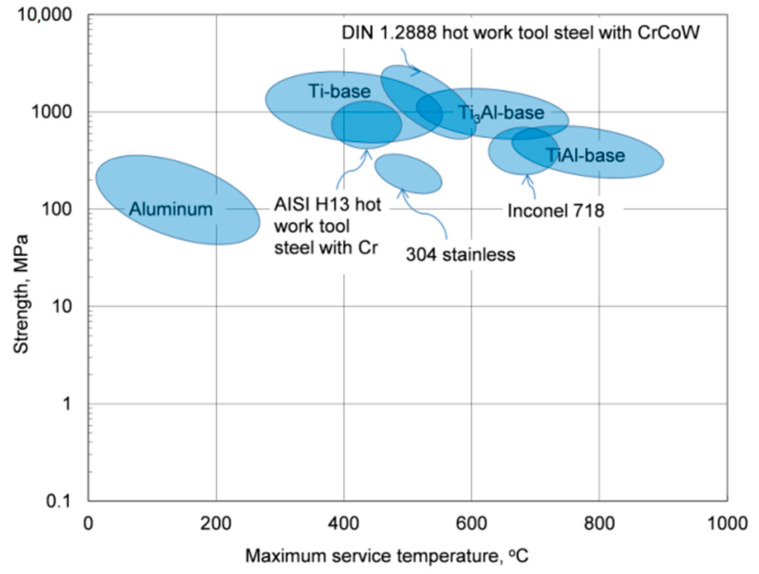
Strength versus maximum service temperature for aluminum alloys and selected structural materials.

Due to the low melting point of aluminum, 660.5 °C, the thermal stability of its alloys covers the temperature range, which is substantially lower than that of other materials with much higher melting points that, excluding the corrosive factor, can be used to contain molten aluminum alloys, as schematically marked in Figure 1. In this respect, the term “heat-resistant alloys” also has a relative meaning when applied to aluminum-based grades.

**Figure 2:**
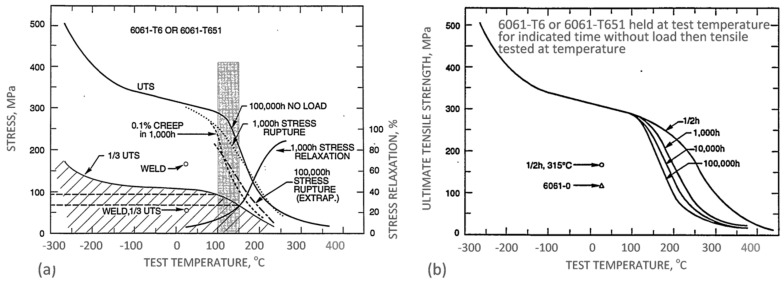
Example of property deterioration of the AA6061 aluminum alloy upon heating in air: (**a**) effect of temperature on creep and stress relaxation; (**b**) effect of temperature and time on ultimate strength [4].

The property variation of aluminum alloys at temperatures from cryogenic to over 400 °C is different than that observed in other materials, such as steel. As for other materials, the intensity of the temperature-related property change of aluminum alloys is influenced by their chemical composition and initial microstructure, controlled, in turn, by the manufacturing route and post-manufacturing treatment. An example for the wrought AA6061 alloy in T6/T651 condition is shown in Figure 2a,b. At temperatures above 150 °C, the alloy suffers a loss in strength with deterioration increasing over time. Above 200 °C, the weakening is substantial, and is accompanied by some gain in ductility. Most of the strength reduction induced by exposure to elevated temperatures is permanent, so the loss in strength is not recovered when the material is returned to a lower temperature. In case of AA6061, a major portion of the loss in strength is caused by coarsening of the Mg_2_Si precipitates. As shown in Figure 2a, aluminum alloys are susceptible to creep and stress relaxation. Creep is a time-dependent, permanent deformation that occurs under sustained load or stress, even at stresses below the yield strength. For the most part, creep is governed by migration of vacant lattice sites, which increases with temperature.

### 2.1. Thermal Stability as a Component Design Criterion: Combined Influence of Temperature and Time

**Figure 3:**
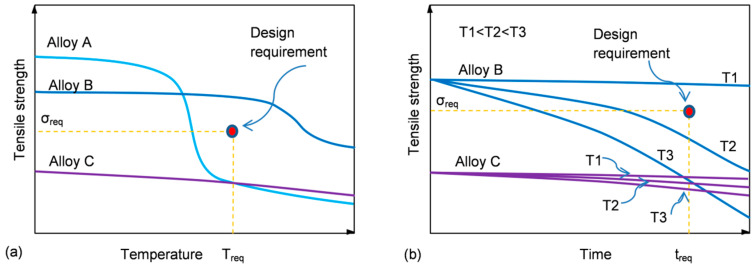
Thermal stability of structural materials and design requirements: (**a**) schematics of hypothetical changes in tensile strength versus temperature (**b**) and strength versus time at constant temperature (**b**), along with the strength required σ_req_ at temperature T_req_ during service time t_req._

In order to apply a material for a particular design, the certain threshold of properties at service temperature is required. To understand the process of material selection, three hypothetical alloys with different strength vs. temperature/time characteristics are shown schematically in Figure 3a,b.

Alloy A has an initial strength that substantially exceeds the minimum required but experiences a steep reduction at temperatures much lower than that predicted in its design (Figure 3a). In contrast, alloy C shows high thermal stability with low reduction in strength, taking place within the entire temperature range. However, its overall low strength makes it not suitable for this application. Thus, from a temperature criterion alone, alloy B meets the design specification.

At a given temperature, the material properties are affected by the exposure time. The influence of time on the properties of aluminum alloys depends on temperature. At high temperatures, a reduction of strength is the dominant observation for all classes of alloys. In contrast, at room or slightly elevated temperatures the opposite behavior may be observed, where the alloy strength may increase at the cost of plasticity, so an alloy may become prone to brittle cracking. The alloy selection depends, therefore, on the kinetics of the strength variation, and for alloy B, the strength reduction at temperatures T2 and T3 makes it not suitable for that design.

As portrayed in Figure 3a,b, a definition of thermal stability as “strength (property) retention at service temperature/time” can lead to confusion during a material selection. Therefore, the highly thermally stable alloy C does not meet design requirements due to its overall low strength. Thus, the thermal stability criterion that is viable during a material selection has two factors: (i) an alloy should achieve at room temperature the strength required and (ii) the strength should be retained within the temperature and time space to meet the level required at service temperature and to remain stable for the predicted service time.

Thermal stability is often expressed through graphs of the alloy strength vs. maximum service temperature. While being very educational, the above examples show that without specifying detailed conditions (time, load, environment, etc.), such characteristics are very approximate.

### 2.2. Understanding the Temperature—Load Factors

The influence of heat on material properties depends not only on temperature but also on temperature changes with time, especially in the case of frequent (periodic) changes, a presence of load applied to a material in structural applications and its nature.

#### 2.2.1. Thermal Exposure—Stable Temperature

**Figure 4:**
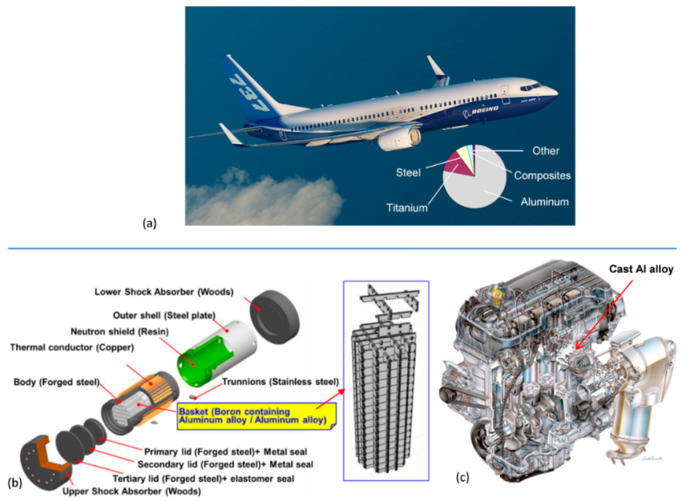
Applications of aluminum alloys requiring thermal stability at different temperatures: (**a**) commercial airplane with aluminum surface components exposed to room and slightly elevated temperatures [5]; (**b**) packaging and transportation of radioactive materials: long-term storage/transportation casks for spent fuels in nuclear power plants, requiring exposure to temperature 100–200 °C for over 60 years [6]; and (**c**) automotive combustion engine with new designs requiring temperature over 200 °C [7].

In general considerations of thermal stability, it is assumed that a material is exposed to constant (or near constant) temperature. As thermal stability refers to all temperature ranges that also include room environment the term “high thermal stability” may often be misleading, when temperature is not specified. For example, an aluminum alloy may be described as having very high thermal stability just at 100 °C. Examples of applications of aluminum alloys that require thermal stability at essentially different temperatures are shown in Figure 4a–c.

Stability at room and slightly elevated temperatures refers to temperatures typically below 100 °C. Such a scenario occurs for aircraft components that may become warm during service due to exposure to sun, aerodynamic heating, or heat transferred from engines. Temperatures from 70 °C to 85 °C are typically used to simulate the environment to which the wings and fuselage structures of a commercial aircraft are exposed [8].

The medium temperature range, requiring thermal stability, covers temperatures below 200 °C. They include impellers used in generators/compressors, vacuum pump rotors, and turbocharger impellers of various sizes with service range of 150 to 180 °C. An example of alloy used in this environment is the Al−Cu−Mg−Fe−Ni AA2618 alloy [9]. The medium temperature range will also cover alloys in metastable state, cold-deformed, nanocrystalline, and amorphous ones. However, the most challenging aspect of thermal stability is maximizing the upper service limit of high-temperature grades with the ultimate goal for aluminum alloys being to exceed 400 °C.

Thermal stability is uniquely tested in a case of accidental fire, when aluminum alloys may be exposed to temperatures, exceeding those predicted for regular service that exert damaging effect on their properties. There is a concern of fire safety with using aluminum for load-bearing applications such as lightweight structures, light rail, bridge decks, marine crafts, and off-shore platforms, due to potential dangerous reduction in mechanical properties during exposure to heating [10]. Alloys used in these applications are typically not designed for high temperatures. A related concern is regarding the integrity and stability of an aluminum structure following a fire exposure.

#### 2.2.2. Constant versus Variable (Cyclic) Temperature—Thermal Fatigue

Due to thermal exposures, materials expand during heating and contract during cooling. When a material is geometrically constrained, this leads to generation of tensile and compressive stresses. Stresses may also arise within unconstrained materials due to a spatial temperature gradient. As a result of cyclic expansion and contractions the material experiences thermal fatigue. This phenomenon, also called heat checking, is common when a metal surface is repeatedly heated and cooled. Thus, thermal fatigue may occur without mechanical loads. If both thermal and mechanical strain is involved, the degradation mode is termed as thermomechanical fatigue.

#### 2.2.3. Role of Load in Thermal Stability—Thermal versus Thermomechanical Response

As the structural materials are subjected to a load at service temperature, the effect of heat on their performance depends on the load level and its nature, with a special impact being exerted by heavy and cyclic loads, in particular with high-speed load alterations. To describe a material performance under particular service conditions the term durability is often used, understood as the ability of a material to sustain mechanical or thermomechanical loads over a predicted service time. Although the durability meaning may vary, depending on an application, for structural materials it is seen as the critical design consideration.

### 2.3. Environmental Effect on Thermal Stability

The ability of retaining the properties by an alloy is strongly affected by reactive environments leading, for example, to oxidation, erosion, molten metal or salt corrosion, or irradiation damage.

#### 2.3.1. Surface Deterioration

For room temperature service, surface corrosion, leading to localized reaction and a material loss, forming pitting and stress risers, is of concern. For service at high temperatures, a process of selective oxidation resulting in localized surface degradation should be considered.

The oxidation of aluminum in air can be described as occurring in four distinct stages [11]. At room and lower temperatures, the amorphous alumina layer covers the metallic surface, protecting it against further oxidation, which results in very slow film thickening up to 550 °C. At this stage, the oxide growth is controlled by outward diffusion of Al ions with a reaction taking place at the oxide−gas interface. The amorphous oxide remains stable, due to the energy of the oxide–metal interface, only up to a critical thickness of ~5 nm, then transforms to γ-alumina, when crystallites are no longer able to form a continuous layer that would cover the aluminum surface. This leads to stage II, above 550 °C, with higher oxidation rate and polycrystalline layer of γ-alumina covering the entire aluminum surface. At the stage III, which starts at 650 °C, very close to melting, the growth of γ-alumina continues at a rate controlled by the inward diffusion of oxygen anions along oxide grain boundaries, acting as fast diffusion paths.

In the case of aluminum alloys, the process of high-temperature oxidation may have a preferential nature, leading to localized oxide patches, formed on specific alloy phases, potentially forming stress risers. The role of aluminum surface reactivity is better understood when compared with another light metal, magnesium. Due to the high affinity of magnesium with oxygen, at high temperatures, its surface degradation and formation of MgO is of higher concern than a reduction in its mechanical properties [12,13]. In this regards, aluminum shows an advantage over magnesium. In contrast to magnesium, there is no concern of ignition or flammability with aluminum and its oxidation rate is substantially slower due to a formation of the protective Al_2_O_3_ alumina film. However, for Al−4−5Mg (wt.%), only MgO is formed with a reaction following the linear law up to 500 °C and parabolic law above 550 °C [14,15]. Then, an environment may essentially change the oxidation kinetics. For example, in a presence of traces of sulfur, spallation of otherwise protective alumina occurs, which is the chronic problem in some aerospace applications.

#### 2.3.2. Irradiation Damage

An important environmental factor, necessary to consider during analysis of thermal stability of aluminum, is the influence of radiation. Aluminum alloys are used in applications subjected to irradiation, e.g., as the primary structural material for the reactor reflector vessel of Advanced Neutron Source and for most of the components housed within the vessel [4] or in casks for transportation of nuclear fuels [16]. The deciding factors for the use of an alloy are good combination of low neutron absorption cross section and high thermal conductivity, good resistance to aqueous corrosion, and good performance in high flux reactors. Therefore, changes in mechanical properties expected to occur in alloys under irradiation during their intended lifetimes are of key importance.

The effects of irradiation exposures result in an increase in the metal’s volume, caused by development of voids, bubbles, and low-density phases, termed as swelling. Although generally metals undergo hardening during irradiation, softening is also possible, particularly for cold work-hardened or precipitation-hardened alloys, subjected to irradiation. That softening may take place during irradiation at temperatures below the normal temperature for thermal recovery, as a result of radiation enhanced diffusion processes or cascade dissolution of precipitates.

**Figure 5:**
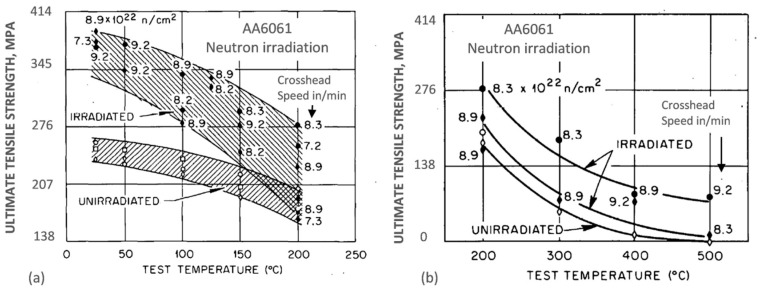
Effect of neutron irradiation on thermal stability of the AA6061 aluminum alloy at temperatures below 200 °C (**a**) and above 200 °C up to 500 °C (**b**) [17].

An example for the AA6061 aluminum alloy target holder from the High Flux Isotope Reactor, originally in a precipitation-hardened condition, after exposure to a maximum fast neutron fluence of 9.2 × 10^22^ neutrons/cm^2^ (E > 0.1 MeV) and a thermal fluence of 1.38 × 10^23^ neutrons/cm^2^ (E < 0.414 eV) at ~60 °C, is shown in Figure 5a,b [17]. At temperatures in the range from 25 to 200 °C, significant strength increases were observed that are attributed to the silicon precipitates and to irradiation-induced dislocations. There was a corresponding loss of ductility, particularly severe at 200 °C for slow strain rate testing conditions. The alloy, tested at temperatures between 200 and 500 °C, at which the microstructure was unstable, showed substantial loss in strength, accompanied by a gain in ductility. For the entire temperature range from 25 to 500 °C, however, the strength of irradiated alloy was higher than that for the alloy without irradiation. The same nature of changes was reported for the AA4043 alloy welds.

In another example, a microwave radiation influenced the thermal stability of aluminum nanosize powder. After microwave radiation with a power flux density of 80 W/cm^2^ and carrier frequency of 9.4 GHz, the chemical activity of aluminum powder increased and the temperature for the beginning of its oxidation decreased by 40 °C, while the thermal effect of oxidation decreased by 13.5% [18].

## 3. Thermal Deterioration of Alloy Strengthening and Testing Techniques of Thermal Stability

When operating temperatures of alloys are increasing, additional problems to those encountered at room temperatures arise. Therefore, to improve the thermal stability of modern alloys, understanding the complex relationships between the alloy chemical composition, processing, microstructure, and properties at service temperature is paramount. It is believed that progress achieved in this area within the last few decades was mostly associated with increased understanding of this relationship.

Alloying elements, when added to aluminum, may generate effects of precipitation hardening (age hardening), solid solution hardening, dispersion strengthening, grain refining, modifying metallic and intermetallic phases, suppression of grain growth at elevated temperatures, wear resistance, and other surface-controlled behavior.

**Figure 6:**
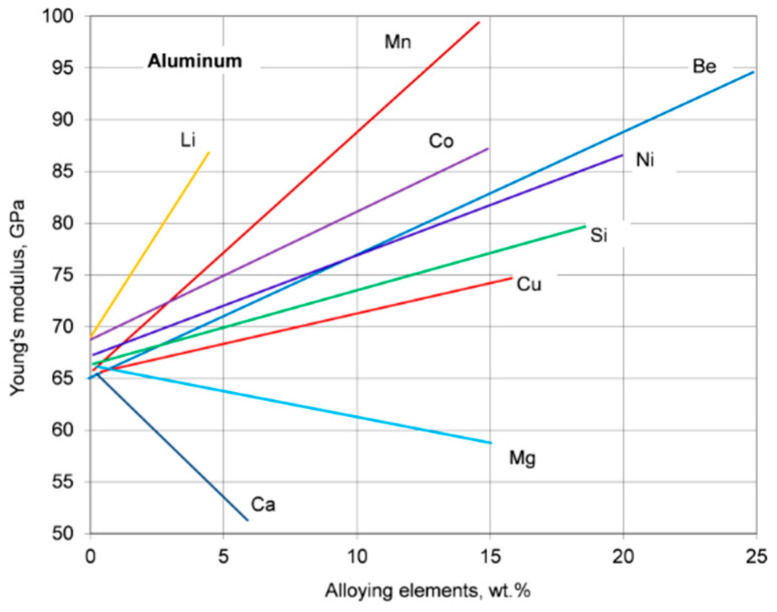
Effect of solute additions on the elastic modulus of aluminum alloys; created based on the work in [19].

The elastic properties of materials can be used to assess certain mechanical properties such as ductility/toughness, hardness, or strength. The Young’s modulus of aluminum may be increased through alloying with the effect being controlled by the element content and its form in the alloy. The effect of solute atoms of typical alloying elements on Young’s modulus of aluminum is shown in Figure 6. For alloying elements, being in a solid solution, a magnitude of the Young’s modulus is determined by the nature of atomic interactions. For alloying elements, forming the second phases, the magnitude of the Young’s modulus is determined by the volume fraction and the intrinsic modulus of those phases.

### 3.1. Alloy Strengthening Mechanisms

Thermal stability relies on changes within an alloy microstructure that are caused by diffusion of alloying elements. During heating, therefore, microstructure components, such as the grain size, phase composition, phase morphology, or solute in the matrix, will evolve. In order to assess the microstructural changes affecting thermal stability, the elementary strengthening mechanisms that act in aluminum should be considered. When an alloy is subjected to heat, the strengthening mechanisms that acted at room temperature will change their effectiveness and contribution to overall alloy strength, measured at high temperature.

#### 3.1.1. Solid Solution Strengthening by Atoms Dissolved in the Matrix

The solute atoms affect the alloy strength by imposing lattice strains on the surrounding host atoms, thus decreasing the dislocation movement and the lattice strain field interactions between dislocations and them. Alloying atoms has a tendency to diffuse and segregate around dislocations to find atomic sites more suited to their radii, which decreases the entire strain energy and immobilize (pin) dislocations. The strength from solid solution hardening arises from strain fields around each solute atom, dependent on its misfit radii. The solution strengthening factors include the relative size factor, relative modulus factor, electrical interaction, chemical interaction, and configurational interaction. Among them, the relative size factor ε_a_ is expressed as [20,21]:

(1) $$\varepsilon_{a}=\frac{1}{a}\frac{da}{dc}$$

where *a* is the interatomic spacing of the alloy and *c* is interatomic concentration of the solute.

#### 3.1.2. Strain Hardening or Cold Working, Caused by Plastic Deformation

Strain or work hardening refers to an increase in stress with strain required to continue plastic deformation. As a material is deformed at low homologous temperatures (below recrystallization), dislocation density increases inside its structure. Aluminum, which is characterized by the high stacking fault energy, deforms by dislocation-mediated slip. The process of strain/work hardening is often portrayed as a competition between the dislocation accumulation and dislocation annihilation, described as dynamic recovery. The basic equation that relates flow stress (strain hardening) σ to the structure is given as [20]:

(2) $$\sigma =\sigma_{i}+\alpha Gb\rho^{1/2}$$

where *σ_i_* is the friction stress opposing motion of dislocation, *α* is a geometrical constant of the order 0.3, *G* is the shear modulus, *b* = 0.286 nm is the Burgers vector for aluminum, and ρ is the dislocation density.

#### 3.1.3. Strengthening by Fine Particles

This strengthening mechanism involves precipitation strengthening or age hardening, resulting from heat treatment and dispersion strengthening after inclusion of dispersoid particles, acting as obstacles to dislocation movement. In an alloy, particles may be cut by dislocations or resist cutting, thereby forcing dislocations to bypass them. In the latter case, the gliding dislocations circumvent dispersoids by the Orowan bypass mechanism and the stress σ at room temperature for idealized spherical particles with radius *r* can be calculated by the Orowan−Ashby equation [20]:

(3) $$\sigma =0.13\frac{Gb}{\lambda }\ln\frac{r}{b}$$

where *G* is the shear modulus, *b* is the Burgers vector, and *λ* is the dispersoid spacing on a slip plane.

#### 3.1.4. Grain Size Strengthening

When the material is deformed and the resistance to plastic flow is controlled by the dislocation glide and diffusion-controlled processes are not an issue, a decrease in the grain size causes the strengthening effect. In this case, the yield stress *σ_gb_* is related to the grain size, d, through the Hall−Petch equation, expressed as

(4) $$\sigma_{gb}=\sigma_{o}+kd^{-1/2}$$

where *σ_o_* is a material constant for the starting stress for dislocation movement or the resistance of the lattice to dislocation motion and k is the strengthening coefficient, specific to aluminum.

Another approach to grain boundary strengthening *σ_gb_* in aluminum alloys is presented by the Nes−Marthinsen formula [22]:

(5) $$\sigma_{gb}=\alpha Gbd^{-1}$$

where *α* is a constant and the other symbols are as in Equation (3). An explanation of the theoretical background behind Equation (5) is available in [22].

**Figure 7:**
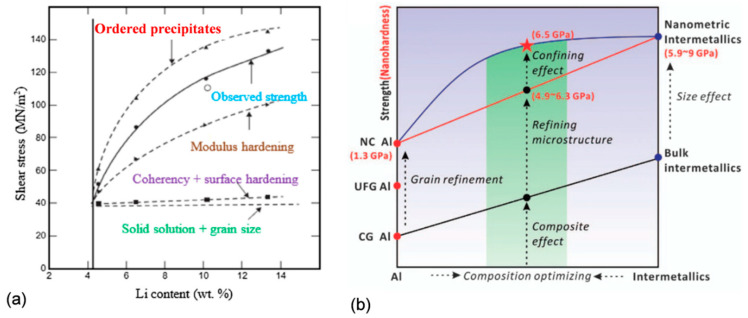
Contribution of different strengthening mechanisms in various aluminum alloys: (**a**) Al−Li alloys with Al_3_Li precipitates [26,27] and (**b**) Al84Ni7Gd6Co3 (at.%) alloy, produced by a combination of gasification, ball milling, and hot pressing [25].

In practice, there are no alloys with a single strengthening mechanism, and during service several contributors simultaneously control the alloy behavior [23]. As shown in Figure 7a, for Al−Li alloys, the solution of Li in the Al matrix is the key contributor and the major strengthening is coming from high volume of Al_3_Li precipitates. Strengthening by the Al_3_Li phase is caused, in turn, by several mechanisms, such as coherency and surface hardening, modulus hardening, and strengthening by coherent ordered precipitates [24]. In strengthening of the Al84Ni7Gd6Co3 (at.%) alloy shown in Figure 7b, the nanoscale Al grain and large volume fraction of intermetallic phases contribute through the composite effect. A lack of fine precipitates in the microstructure results in negligible Orowan strengthening [25].

For cold-worked AA1050 alloy, where precipitation hardening is not involved, the contribution from grain boundary strengthening was ~30 MPa, with the remaining yield stress of 170 MPa being covered by dislocation strengthening [28]. In the complex microstructure of an extruded AA7075 alloy, the major strengthening contributors consisted of grain boundary, dislocation, solid solution, precipitation, and oxide dispersoid strengthening [29]. As temperature increases, the individual strengthening factors will selectively change their effectiveness. For example, in amorphous alloys, crystallization and formation of fine precipitates will represent key changes. For nanocrystalline alloys, grain growth will be of primary concern, when temperature increases.

### 3.2. Microstructure Deterioration under Thermal Exposure

#### 3.2.1. Diffusivity of Alloying Elements in Aluminum

**Figure 8:**
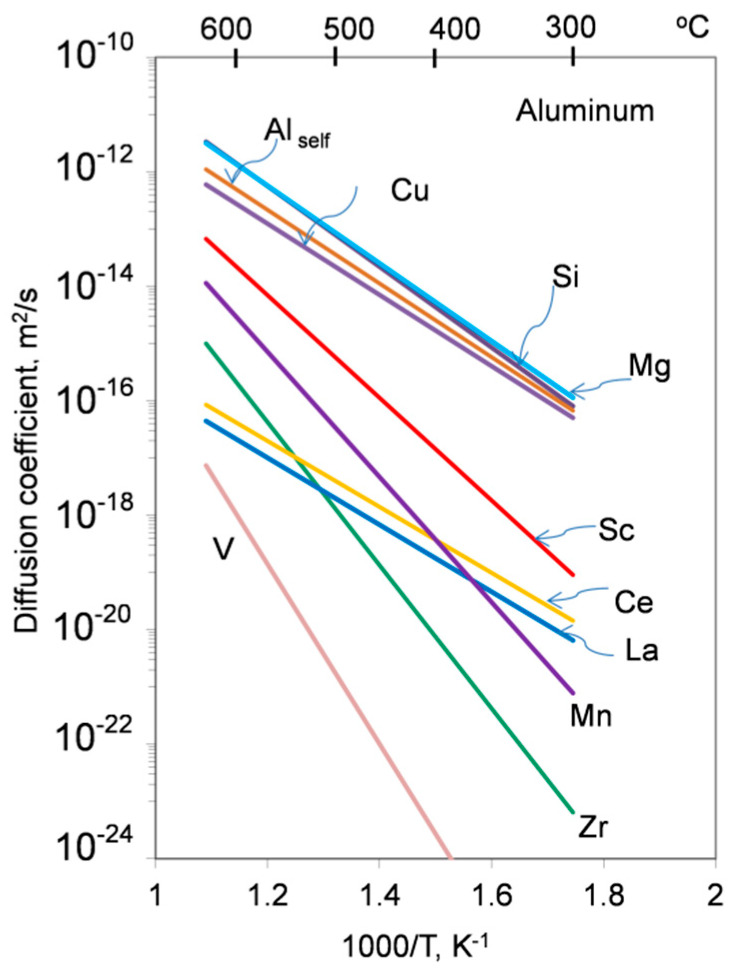
Semi-logarithmic plot of diffusivity in aluminum versus reciprocal temperature for alloying elements, representing transition metals, rare earth and non-transition metals, along with aluminum self-diffusion. Plot based on data selected from Table A1, Table A2 and Table A3 in Appendix A.

**Figure 9:**
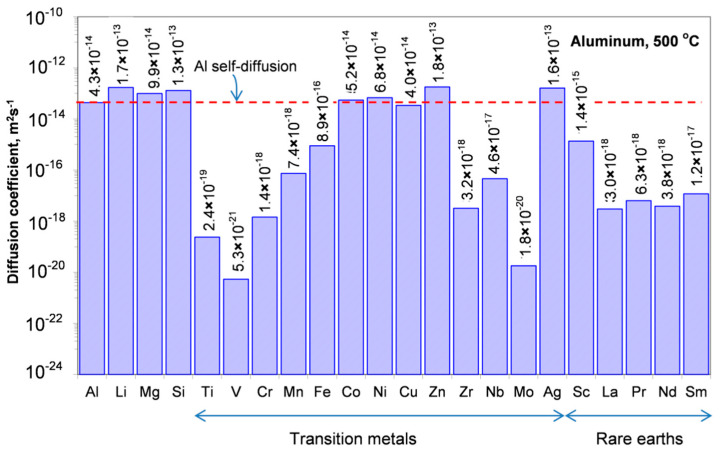
Calculated diffusion coefficients at 500 °C of selected alloying elements in aluminum, representing transition metals, rare earth and general elements, along with aluminum self-diffusion. Plot based on data from Table A1, Table A2 and Table A3 in Appendix A.

The diffusivity of alloying elements is the main indicator of the alloy stability at a service temperature. Diffusion, which for substitutional elements is enabled via mobile atomic lattice vacancies, often requires nonequilibrium vacancies to cause structural changes. Aluminum is the trivalent fcc metal with an impurity diffusion, being essentially different than that encountered in the monovalent and fcc noble metals. As evidenced for impurity diffusion, elements with low solid solubility in Al have diffusion coefficient low as compared with Al self-diffusion. A comparison of the diffusivities of selected alloying elements in Al is shown in Figure 8 and Figure 9.

Throughout this report, the classification recommended by International Union of Pure and Applied Chemistry [30] is used, where rare earths elements are seen as a series of 17 elements: scandium, yttrium, and the lanthanide series (lanthanum, cerium, praseodymium, neodymium, promethium, samarium, europium, gadolinium, terbium, dysprosium, holmium, erbium, thulium, ytterbium, and lutetium).

According to alternative classification [31], rare earth elements are seen as lanthanides only and scandium with yttrium along with other 36 elements of Groups III through XII are classified as transition metals. Cerium belongs to Light Rare Earth Elements (LREE) also known as the cerium group (Sc, La, Ce, Pr, Nd, Pm, Sm, Eu, and Gd). The second group—Heavy Rare Earth Elements (HREE)—is known as the yttrium group (Y, Tb, Dy, Ho, Er, Tm, Yb, and Lu).

##### Diffusion of General Elements and Self-diffusion of Al

Aluminum preserves its fcc structure up to the melting temperature. The self-diffusion of aluminum has been measured by combining various techniques, including radiotracer with 26Al [32], Nuclear Magnetic Resonance Spectroscopy [33], and TEM observation of voids shrinkage [34].

General alloying elements have diffusivities slightly higher than the aluminum self-diffusion with only small variations among different elements (Appendix A, Table A1). Their activation enthalpies are similar to those of aluminum self-diffusion and almost independent of their valence. The key element in this group, Si, diffuses faster than Al in Al−Si alloys, as proven during determination of diffusion coefficients in Al−Si alloys by Matano’s method, where Kirkendall markers were found to move toward the Si-rich side [35].

##### Diffusion of Transition Metals in Aluminum

The majority of transition metals have diffusivities lower than the aluminum self-diffusion (Appendix A, Table A2). In general, they have high activation enthalpies and they also have high pre-exponential factors. High activation barriers and anomalously low diffusion coefficients, for partially filled d−shell 3d elements, reflect strong bonding of 3d elements with Al. Exceptions to this are Co and Ni with high d−d interactions, and thus weak bonding with Al [36].

Similar diffusivity as Al self-diffusion is shown by Co, Ni, and Cu; higher diffusivity is shown Zn and Ag but lower diffusivities have Fe, Mn, Cr, Ti, V, Hf, Mo, and Pd [37,38].

##### Diffusion of Rare-earth Metals in Aluminum

Rare earth metals have very low solid state solubility in aluminum, large atomic radii and ionic charge of three—the same as aluminum. Unfortunately, the diffusion data for rare earth elements in aluminum are limited, with the work in [39] from the 1960s still being the major source (Appendix A, Table A3). In general, their diffusion coefficients are up to 4 orders of magnitude lower than Al self-diffusion. An exception is Sc, sitting in the midway between the rare earths and aluminum (Figure 8 and Figure 9).

#### 3.2.2. Coarsening Resistance of Precipitates

**Figure 10:**
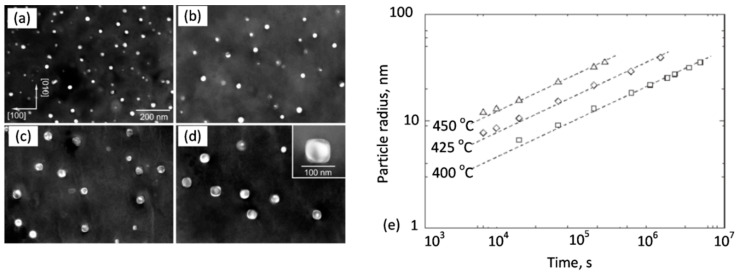
Precipitate coarsening during aging of Al−0.28 wt.% Sc alloy: dark-field TEM images of Al_3_Sc precipitates aged at (**a**) 400 °C for 5 h, (**b**) 400 °C for 50 h, (**c**) 425 °C for 50 h, and (**d**) 450 °C for 50 h; (**e**) change in the average precipitate radius *r* with aging time *t* for temperatures indicated [41].

The essential factor that preserves the alloy properties at high temperatures is dispersed strengthening particles, resistant to diffusion-controlled coarsening, called Ostwald ripening. This coarsening mechanism, with an example shown in Figure 10, is governed by the Gibbs−Thompson effect, which alters the concentration at the particle−matrix interface, depending on the curvature of the interface. As a result of the concentration gradient, solutes diffuse from small particles to larger ones, leading to the coarsening phenomenon. With increasing temperature, large particles tend to grow at the expense of small ones, the average particle size increases and the total number of particles decreases. Through Ostwald ripening, the system may reduce its interfacial area, and thus its energy. The concentration at the particle−matrix interface is expressed by [40]:

(6) $$C_{A}\left(R\right)-\;C_{A}\left(\infty \right)=\frac{\alpha }{R}$$

where *C_A_*(*R*)—composition of the matrix phase A outside of a particle with radius R; *C_A_*(∞)—composition of the matrix phase A at equilibrium, when the interface is flat; and α—constant, proportional to the specific interfacial energy.

The classical interpretation (LSW) of the Ostwald ripening was developed by Lifshitz and Slyozov [42] and Wagner [43] during examination of coarsening in a system, where globular particles are indefinitely separated. According to the LSW theory, under steady-state conditions the cube of the average particle radius increases linearly with time:

(R(t))^3^ − (R(t))^3^ = K_LSW_ t (7)

where R(0) and R(t) represent the average radius at time t = 0 and t = t, respectively, and K_LSW_ coarsening rate constant expressed as [44]:

(8) $$K_{LSW}=\frac{8\gamma DC\infty vm}{9RT}$$

where *γ* is the interface energy, *D*—apparent diffusion coefficient of solute atoms, *C*∞ the solute concentration at equilibrium,* v_m_*—solute molar volume, *R* is the gas constant, and *T*—temperature. In the *LSW* theory the diffusional interactions between particles are neglected so the mechanism is applicable in a negligible fraction of the coarsening phase.

### 3.3. Testing Techniques of Thermal Stability

The thermal stability of an alloy consists of many components that individually also depend on temperature. For example, thermal stability of microstructure, which, in turn, will impose thermal stability of phase composition, which will further separate into thermal stability of phase size (resistance to coarsening), chemical stability of individual phases, etc.

#### 3.3.1. Microstructure Analysis

Microstructural observations are essential in assessing thermal stability. During conventional imaging, samples are heated ex situ to different stages and subsequently inspected one by one at room temperature. In contrast, in situ TEM allows direct observations of microstructural changes on the nanometer-scale, occurring for all precipitates in a selected sample region. For example, in situ TEM analysis was found effective in assessing thermal stability of the AA5456−H116 Al–Mg alloy after ultrasonic impact treatment [45]. Based on TEM observations, the alloy structure was found stable up to 300 °C followed by grain growth, especially intensive above 400 °C.

#### 3.3.2. Thermal Analysis

Thermal analysis is defined as a group of physical/chemical methods that deal with studying materials and processes under conditions of programmed changes of the surrounding temperature. The physical parameters, measured during thermal analysis, include mass, temperature, heat flux or dimension [46]. While the early thermal analysis techniques assessed exclusively the temperature of the examined sample on heating/cooling, during technology development the reference (inert) sample was added, leading to Differential Thermal Analysis. The most common techniques of thermal analysis include Differential Scanning Calorimetry (DSC), Thermogravimetric Analysis (TGA), Dynamic Mechanical Analysis (DMA), Isothermal Calorimetry (TAM), Thermomechanical Analysis (TMA), Flash Diffusivity, Thermal Conductivity, and Dilatometry (DIL).

Thermal analysis methods are sometimes combined to gather complimentary data, e.g., DSC with TGA for studying the oxidation of aluminum [11] or Al−Mg−Er alloy thermal stability [47]. Most often, however, they are used with other experimental techniques, such as metallography, X-ray, or mechanical testing. For example, DSC coupled with tensile tests and TEM was successfully applied to investigate the effect of thermal exposure at 150 °C on microstructure and mechanical properties of the AA 2524 alloy [48].

**Figure 11:**
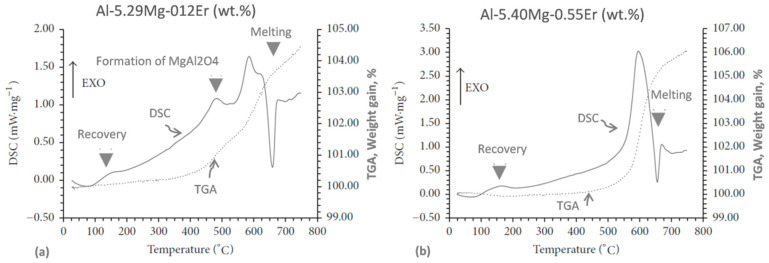
Application of DSC/TGA combination to study thermal stability of Al−Mg−Er alloys: (**a**) Al−5.29Mg−012Er (wt.%); (**b**) Al−5.40Mg−0.55Er (wt.%). The characteristic temperature of solidus being 575 °C and recrystallization at ~330 °C are not detected by DSC [47].

Although very useful, thermal analysis techniques have limitations, as shown in Figure 11 for the cryomilled Al−Mg−Er powders [47]. A combination of DSC and TGA measurements of the nanocrystalline system during heating successfully detected phenomena of melting at ~635 °C (liquidus) and recovery at ~160 °C. However, the solidus temperature, expected at ~575 °C and recrystallization, expected at ~330 °C, as verified by TEM/X ray, were not detected by the DSC/TGA techniques.

#### 3.3.3. High Temperature X-Ray Diffraction

X-ray diffraction (XRD), combined with in situ high-temperature experiments, allows for qualitative/quantitative identification of crystalline phases, with details regarding the crystallite size, texture, lattice parameters, and residual stresses. It allows determining crucial details, such as temperature-dependent phase transformations, thermal changes of structural parameters, and thermal stability of individual phases. High-temperature XRD may measure the changes of physical properties taking place within materials during a simulation of industrial processes.

**Figure 12:**
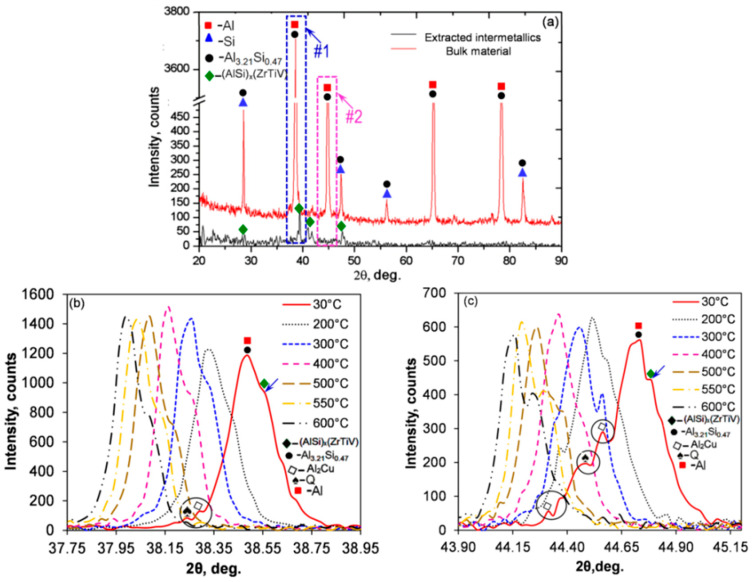
An application of high-temperature X-ray diffraction for thermal stability testing of phases in as-cast Al–7Si–1Cu–0.5 Mg alloy with 0.21Ti, 0.3V, and 0.47Zr (wt.%): (**a**) room temperature diffraction patterns obtained from bulk materials and extracted intermetallics; (**b**,**c**) diffraction patterns at 2θ locations for peaks #1 and #2 at temperatures from 30 to 600 °C. The experiment was conducted with Cu_Kα_ radiation at 45 kV and 40 mA, with a step size of 0.05 deg, and duration of 0.2 s for each step [49].

An example of application of high-temperature XRD to study thermal stability of phases in the Al–7Si–1Cu– 0.5Mg (wt.%) alloy, modified with micro-additions of transition metals is shown in Figure 12 [49]. During heating, the XRD measurements were conducted between 30 and 600 °C, with the particular temperature being selected based on thermal analysis, emphasizing the major transformation points. The experiment concluded that the Cu- and Mg-rich phases along with eutectic Si dissolved between 300 to 500 °C. In contrast, the complex (AlSi)_x_(TiVZr) phases with D0_22_/D0_23_ tetragonal crystal structure and different lattice parameters, containing transition metals and being key to the alloy thermal stability, were stable up to 696–705 °C.

#### 3.3.4. Neutron Diffraction

Neutron diffraction is a form of elastic scattering, where the intensity pattern around the sample irradiated with neutrons allows a determination of its atomic and/or magnetic structure. The technique is similar to described above X-ray diffraction, but the different type of radiation gives complementary information. An advantage of using neutrons lies in the highly penetrating nature of radiation that ensures that the scattering observed is representative of the large material volume. Therefore, it is well suited to measure the thermal stability of alloys subjected to external load.

**Figure 13:**
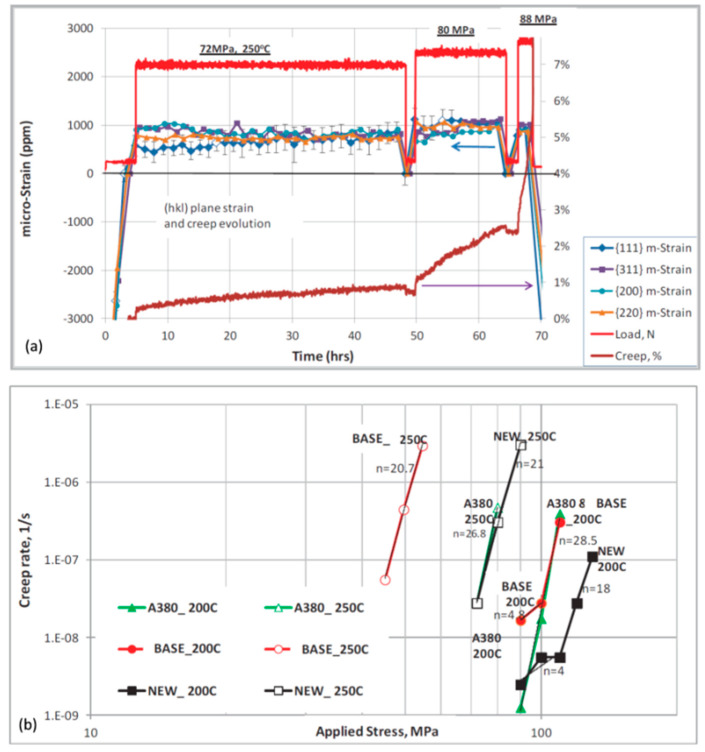
Application of neutron diffraction to the in situ study of thermal stability through measurements of creep evolution: (**a**) creep strain (%) and elastic microstrain (ppm) evolution at selected hkl planes at 250 °C under ascending cyclic loading for Al alloy marked as New below; (**b**) calculated stress exponents of the tested alloys at 200 °C and 250 °C. Alloys: A380: Al9.5Si−3.4Cu, Base: Al−7.1Si−1Cu−0.5Mg, New: Al−7Si−0.9Cu−0.5Mg−0.56Zr−0.20Ti−0.32V (wt.%) [50].

An example in Figure 13 shows an application of in situ neutron diffraction for assessing the influence of temperature on performance of aluminum alloys through measurement of the creep strain for individual *hkl* planes under tensile load [50]. The conditions of temperature and pressure used during measurements are typical to those experienced by automotive engine heads in service. The in situ neutron diffraction measurements provided data on *d* spacing evolution for various *hkl* crystallographic planes as a function of temperature, tensile load, and time, thereby revealing *hkl*-specific evolution of elastic strains. As a result, high temperature plastic and elastic creep properties of alloys were characterized that may be used as a benchmark during development of heat-resistant alloys.

The alloy containing additions of transition metals Ti, Zr, and V shows superior performance under conditions simulating an in-service automotive powertrain component. Its performance was superior in both the ultimate strength and total (plastic and elastic) creep, which can be explained in part by activation all the observed *hkl* planes to support the load. Such findings contribute to in-depth understanding of the structural factors, controlling thermal stability of different alloys under specific load conditions, required in service environment.

#### 3.3.5. Methodology Based on Correlating Hardness and Phase Transformations

**Figure 14:**
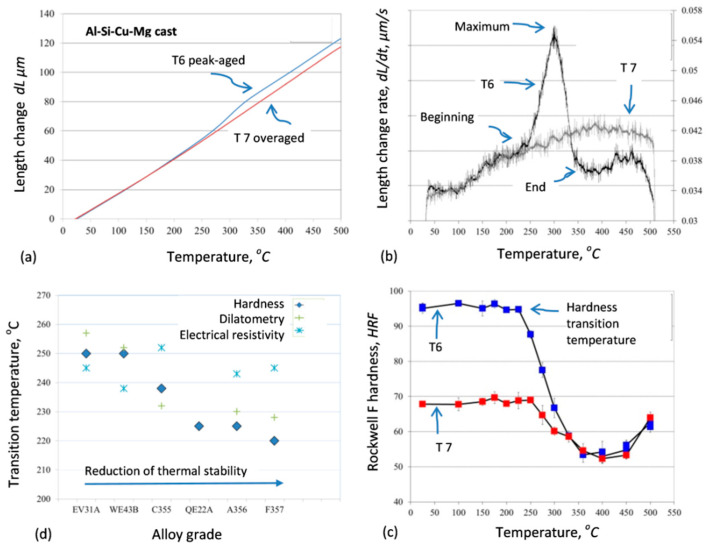
A methodology for determination of alloy thermal stability based on a combination of dilatometry and hardness measurements. Example for cast Al−Si−Cu−Mg alloy in T6 peak-aged and T7 over-aged conditions: (**a**) length change (dL) vs. temperature; (**b**) length change rate (dL/dt) vs. temperature; (**c**) hardness change versus temperature; (**d**) methodology application: thermal stability of commercial aluminum and magnesium alloys determined by hardness, dilatometry, and electrical resistivity measurements [51].

To develop the methodology for assessment of the performance of aluminum alloys at high temperatures, the maximum temperature that alloys can withstand without drastic loss of hardness was determined and correlated with the corresponding phase transformation data [51]. The methodology essence is shown in Figure 14, and the results suggest that the dilatometric measurements in a combination with electrical resistivity experiments represent an efficient and powerful tool to predict the temperature of hardness reduction in commercial aluminum alloys. The methodology was tested on A356 (Al−7.5Si−0.45Mg, wt.%), F357 (Al−7.5Si−0.1Cu−0.5Mg wt.%), and C355 (Al−5Si−1Cu−0.5Mg, wt.%) alloys, being key candidates for a variety of structural applications. During heating up to 500 °C, the alloys experienced from 34 to 66% reduction of the initial hardness with the hardness reduction showing a steep transition between 220 and 238 °C. This methodology is seen useful to determine the suitability of metallic alloys for high-temperature applications in aerospace and automotive industry.

Similar techniques, combining isochronal annealing with microhardness, electrical resistivity, and differential scanning calorimetry, are described in the literature. For example, they were used to assess the effect of small additions of Sc and Zr on microstructure along with thermodynamic and mechanical properties of Al−Sc−Zr alloys [52]. There are also reports on using combinations of dilatometry and electrical resistivity with differential scanning calorimetry for this purpose.

#### 3.3.6. Assessing Thermal Stability through Mechanical Properties

Thermal stability is often visually presented through plots of the alloy hardness versus temperature or versus time at a given temperature. In practice, all mechanical properties, being of importance in engineering applications, experience changes at service temperature. The alloy hardness is commonly used as an indicator of its performance at temperatures up to 500 °C. To determine the aging parameters required to activate the specific type of precipitates and to evaluate the phase transformation kinetics during alloy aging, the isochronal annealing experiments are used, where hardness is the key indicator.

In addition to *hardness* also *strength and ductility* are seen as important design characteristics that define the resistance of an alloy to degradation of ductility and toughness, when subjected to long-term thermal exposure. The tensile or compression testing may be performed at high or room temperature. The routine measurement of thermal stability is the room temperature tensile testing, following high-temperature, often very long-term, exposure.

*Creep strength* and rupture strength are commonly used when designing components for high temperature service. Rupture strength is defined as the stress at specified environmental conditions, such as temperature and a reactive chemical environment, required to produce rupture in a fixed amount of time. It is of interest that while general properties favor the fine grain structures, creep resistance is improved by coarse grains [20].

In some applications of Al alloys, e.g., in automotive engines, the temperature effect is superimposed on cyclic loading, leading to *high-temperature fatigue*. Fatigue failure is defined as a permanent damage of a material due to dynamic loads and may occur at stresses well below the static yield stress. The temperature effect causes the substantial reduction of fatigue life of aluminum alloys. In some applications, the mechanical and thermal strain varies independently, leading to combined, more damaging loading, being termed *thermomechanical fatigue*.

**Figure 15:**
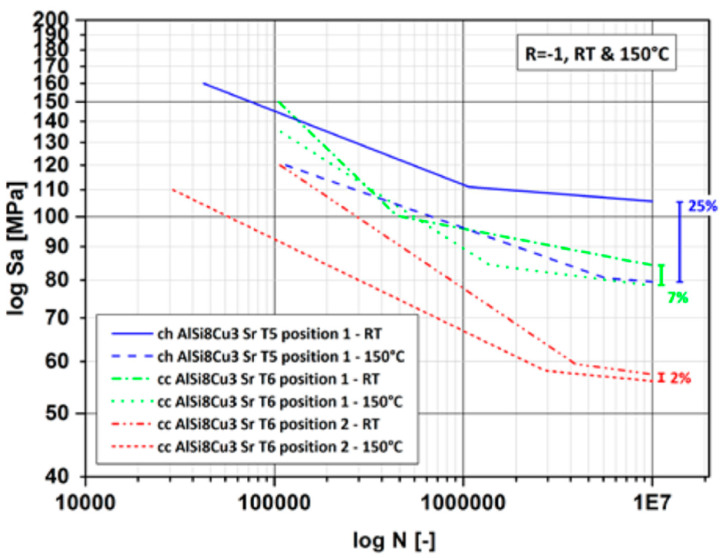
A summary of the stress/cycle S/N curves at room temperature and at 150 °C for a survival probability of 50% of AlSi8Cu3 (wt.%) alloy also listed with the fatigue strength reductions in percentage. It shows that at 150 °C the fatigue strength decreased by up to 25%, depending on location within the part due to porosity; Sa—stress amplitude [53].

An example for the cast AlSi8Cu3 (wt.%) alloy in Figure 15 shows that at 150 °C the fatigue strength decreased by up to 25% for locations within the part, having different local porosity [53]. For the AA7075 alloy, fatigue life reduction factor increased with temperature, reaching a maximum of 44% at 225 °C [54]. For this alloy, a reduction of fatigue life in the temperature range from 25 to 225 °C is described by the Basquin’s equation [55]:

(9) $$\sigma_{f}=\alpha N_{f}^{b}T^{c}$$

where *σ_f_* is the fatigue strength (MPa), *N_f_* is the number of cycles to failure, *α* is the fatigue strength coefficient, and *b* is the Basquin’s exponent. According to the authors of [54], the equation is of the form

(10) $$\sigma_{f}=1558.21N_{f}^{-0.1308}T^{c}$$

where

*c* = 0.54564 − 0.004503 *T* + 1.24567 × 10^−5^*T*^2^ − 1.170266 × 10^−8^*T*^3^ (11)

All the mechanical properties mentioned above are measured with conventional techniques, but with high-temperature capabilities.

#### 3.3.7. Challenges with Predicting the Effect of Long Service Time at Increased Temperatures

When specifying the maximum service temperature of structural materials a distinction is often made between the intermittent and continuous service. It is clear that the temperature of the latter is generally lower. There is an obvious challenge to predict the long-term thermal stability of alloys. The key question is how, within a reasonable laboratory testing time (weeks rather than months), to predict the material future performance over many years, often many decades.

In the area of Ni-based superalloys, facing a similar challenge, a program was initiated in the 1970s to document their long-time thermal stability with testing at 870 °C for up to 16,000 h (almost 2 years) [3]. However, the service time of superalloys, e.g., in turbine blades, is strictly controlled and is much shorter than aluminum alloys applied, for example, in automotive combustion engines.

**Figure 16:**
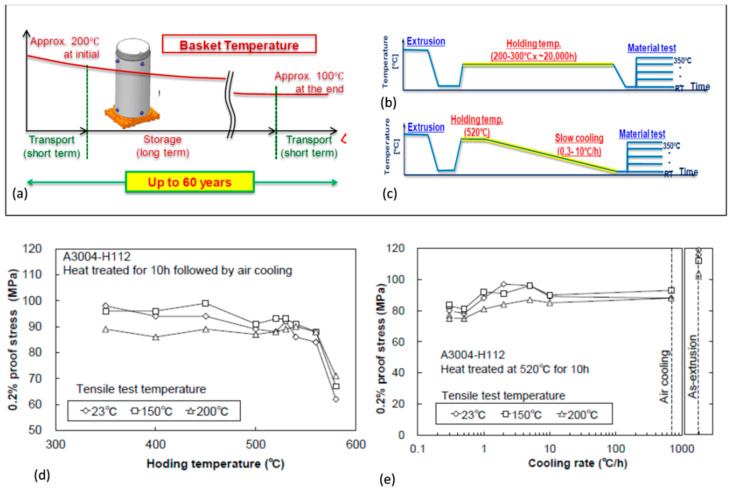
Evaluation of thermal stability of an aluminum alloy for long-term storage/transportation casks for spent fuel in nuclear power plants: (**a**) design requirements of temperature−time exposures; (**b**) long-term holding treatment parameters; (**c**) parameters of annealing and slow cooling treatments; (**d**,**e**) yield stress of the AA3004 H112 extruded alloy after treatment and testing at temperatures indicated [6,16].

An example of an application requiring long-term thermal stability is packaging and transportation of radioactive materials. An aluminum alloy, used for the basket within the storage/transportation casks for spent fuels in nuclear power plants, due to its low density and good thermal stability, should preserve its properties during exposures to temperatures of 100–200 °C for over 60 years [6,16,56]. The cask is used for transportation of spent fuel to a storage facility, where it serves its storage function for up to 60 years, and then it is used again for transportation of the fuel to a spent fuel reprocessing facility or a final disposal site. Throughout its service time the alloy is subjected to temperatures that gradually decrease from 200 °C to approximately 100 °C at the end of 60 years storage (Figure 16a). The alloy should maintain the strength required during storage and transportation, including accidental drop from a height of 9 m, as required by IAEA.

A thermal treatment method was developed to assess the aluminum alloy properties in the basket application, anticipated after long-term storage for 60 years [16]. It consists of two alternate routes: (i) long-term overaging heat treatment at 250 and 300 °C for up to 10^4^ h or (ii) slow cooling from high-temperature annealing at 350 to 580 °C for 10 h (Figure 16b,c). The evaluation of the properties of the AA3004−H112 H112 hot-extruded Al−Mn alloy, Al−1−1.5Mn0.8−1.3Mg (wt.%), after both treatments is shown in Figure 16d,e.

## 4. Alloys Prone to Instability at Room or Slightly Elevated Temperatures

This phenomenon refers to temperatures typically below 100 °C. Such a scenario applies to aircraft components that may become warm during service due to exposure to sun, aerodynamic heating, or heat transferred from engines. Temperatures from 70 to 85 °C are typically used to simulate the environment to which the wings and fuselage structures of commercial aircraft are exposed [8]. For a supersonic jet, due to air friction at a speed of Mach 2.05, the temperature on the skin of the airplane body is reported as 127 °C [57]. A challenging factor is the long exposure time, as airplane materials are designed for a service time between 30 to 50 years. The aerospace applications have strict regulations, regarding thermal stability.

### 4.1. Application of Aluminum Alloys for Aircraft Structure

In today’s aircraft industry, there is a general shift from metallic materials towards composites, but aluminum alloys still represent ~60% by weight of the structural materials used. An exception is the Boeing 787 Dreamliner, which uses only 20% aluminum. Although there are different selection criteria, aluminum alloys are also used in spacecraft structures, including space vehicles and satellites.

The typical alloys used for aircraft structure are Al−Li grades containing lithium to decrease the weight of aluminum, while improving its strength, toughness, corrosion resistance, and forming characteristics. Lithium contents up to 3 wt.% exert a large effect on the modulus of aluminum with a 6% increase for every wt.% Li added. In addition, every wt.% Li added decreases the aluminum density by 3%. Aircraft industry applications include wing leading and trailing edges, fuselage bulkhead webs, and internal framework parts. For example, the Airbus A350 XWB has parts made of steel and titanium, with almost 20% made from Al−Li alloys.

### 4.2. Thermal Stability Concerns with Al−Li Grades

A key question in controlling the thermal stability is how fast the properties evolve, which relates to the kinetics of phase transformations, primarily controlled by diffusion. A room temperature exposure of some aluminum alloys may result in natural aging, i.e., slight movements of solute atoms in the matrix, which modifies the material properties [58]. Aging of Al−Li alloys results in decomposition of solid solution of Li in Al and precipitation of Al_3_Li [59], which may lead to embrittlement during the long-term operation [60,61].

**Figure 17:**
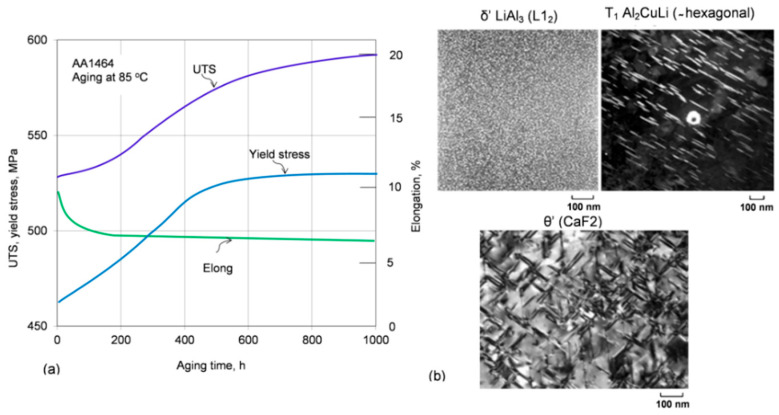
Tensile property change during long-term aging of 2nd generation Al−Li AA1464 alloys at 85 °C, showing an increase in strength and reduction in ductility (**a**) [61] and strengthening precipitates in third generation Al−Li alloys (**b**) [62].

Historically, the first (1950s and 1960s) and second (1980s) generations of Al−Li alloys tended to suffer from several problems, including poor ductility and fracture toughness, fatigue and fracture resistance, and unreliable corrosion resistance [62]. The second generation alloys AA2090, Al−Li−Cu, and AA8090, 2091 Al−Li−Cu−Mg, when exposed to elevated temperature of ~70 °C undergo aging, causing an increase in strength and reduction in ductility and toughness that may lead to embrittlement. An explanation of embrittlement in the AA8090 alloy, considered the most successful of the second generation, includes a formation of δ AlLi phase at grain boundaries, segregation of Li atoms to grain boundaries, precipitation of small Al_3_Li particles, and precipitation effects related to G−P zone formation [63]. An example of property change for the second generation Al−Li alloy AA1464, subjected to long-term exposure at 85 °C and strengthening precipitates in third generation alloys, are shown in Figure 17a,b.

The third generation Al−Li alloys, developed in the 1990s, with significantly reduced lithium content and other improvements, made them more attractive for modern aircraft and aerospace vehicles. For example, the Airbus A380 uses 3rd generation Al−Li alloys AA2099−T83 and AA2196−T8511 for floor beams, AA2196−T8511 for fuselage stringers, and AA2050−T84 for lower wing reinforcement [64]. Thermal stability of the AA2099 Al−Cu−Li T83 alloy, assessed in the temperature range 200 to 305 °C, through both hardness and tensile tests after overaging, showed better performance, compared to aluminum alloys specifically developed for high temperature applications, with the advantage of a considerable lower density [65]. Tests underlined the need to enhance the formation of T_1_ (Al_2_CuLi) precipitates when high temperature strength is required. An addition of 0.29 wt.% Ce contributed to coarsening inhibition of Al_2_CuLi and refinement of Ce-containing intermetallic phase, Al_8_Cu_4_Ce, which further improved the thermal stability at the medium−high temperature range (170 to 270 °C) and high-temperature deformation uniformity of this alloy [66].

The Al–Li–Cu–Zr alloy, C458−T861 (Al−1.8Li−2.7Cu−0.3Mg−0.08Zr−0.3Mn−0.6Zn wt.%), destined for use in space vehicles, tested at 83, 135, and 177 °C for up to 1000 h showed good thermal stability of mechanical properties up to 135 °C. However, further increasing the temperature and time led to a reduction in the alloy fracture toughness [67].

## 5. Stability of Alloys with a Nonequilibrium State

The upper strength limit of bulk Al alloys achieved by conventional precipitation strengthening of ~700 MPa may be increased to over 1000 MPa through grain refinement to nanocrystalline level and amorphization. Unfortunately, the nonequilibrium state with a low thermal stability of amorphous and nanostructured aluminum alloys limits their use at increased temperatures.

### 5.1. Thermal Stability of Amorphous Alloys (Metallic Glasses)

Al-based amorphous alloys represent an important group of amorphous materials with a high specific strength, combined with outstanding corrosion resistance and good ductility. It is of interest that their high strength at room temperature is accompanied by an ability of softening to viscous liquid states above the glass transition temperature, thus allowing thermoplastic forming to be conducted due to superplasticity [68].

In the 1970s, the alloys covered Al−transition metal binary systems, formed by splat quenching, and in the 1980s, the Al−(Fe, Co)−B alloys, cast by melt spinning. Later, they expanded to alloying with transition metals groups IV−VI with VII and VIII. The next expansion included Al−rare earth binary systems and Al−transition metals−rare earth combinations. The latter systems are the most popular at present due their high glass-forming ability and high strength. There are still technological barriers with manufacturing the Al-based bulk metallic glasses due to their rather low glass-forming ability, requiring very high critical cooling rates that are difficult to achieve in engineering practice at larger cross sections, often larger than just a few micrometers.

During heating, amorphous alloys easily transform to crystalline structures, losing their unique properties. Following crystallization, continued heating results in phase transformations with a generation of stable and metastable phases, until the alloy finally melts. For example, amorphous powders with compositions of Al85Y7Fe8, Al83Y7Fe8Ti2, and Al79Y7Fe8Ni3Ti2Nd1 (at.%) exhibited crystallization temperatures of 342, 446, and 457 °C, respectively, with an increase by 115 °C through microalloying [69].

**Figure 18:**
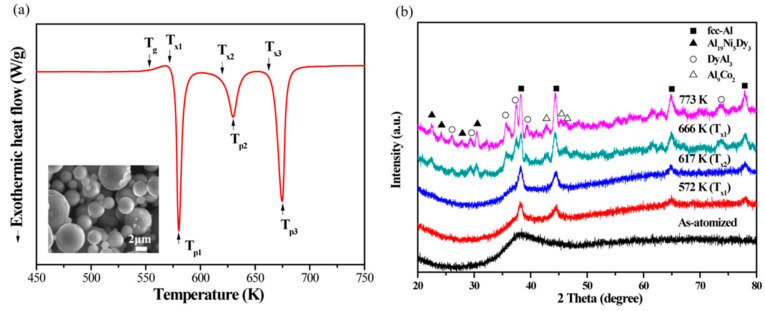
Thermal stability of amorphous Al84Ni7Co3Dy6 alloy in gas-atomized powder form: (**a**) DSC trace obtained during continuous heating of at a heating rate of 40 K/min with inset showing the powder morphology; (**b**) XRD patterns after heating to 299, 344, 393, and 500 °C. Both techniques show crystallization phenomena of marked phases [70].

An interesting feature of the crystallization behavior of amorphous aluminum alloys is that some compositions have a tendency to crystallize into nanometer-sized clusters or grains (Figure 18). The phenomenon can be explored for improving properties of amorphous structures. For example, the mechanical properties of bulk Al84Ni7Co3Dy6 (at.%) alloys, produced by Spark Plasma Sintering of amorphous powder with a diameter of 25 µm at temperatures above 400 °C, are significantly enhanced by in situ crystallization of nanoscale intermetallic compounds during plasma sintering [70]. The Al−Ni−Co−Dy bulk alloys produced by plasma sintering at 400 °C exhibit a maximum strength of 1773 MPa with 5.6% plastic strain. Increasing the sintering temperature to 430 °C led to higher plastic deformability of 7.2% at the expense of the lower maximum strength of 1255 MPa. The solute concentration played a key role in determining the size of the α−Al phase during plasma sintering of the amorphous Al84Ni7Co3Dy6 (at.%) powder.

### 5.2. Thermal Stability of Nanocrystalline Alloys

Nanocrystalline materials have a high stored energy due to their large grain boundary area and, when subjected to heating, grains have a tendency to grow to minimize their energy by reducing the grain boundary area per unit volume [71]. The poor thermal stability of nanocrystalline materials arises from their high density of grain boundaries, which provides a high driving force for grain coarsening. Overall strategies that are proposed to stabilize the grain size with emphasis on thermodynamic stabilization and kinetic stabilization are discussed in [72].

**Figure 19:**
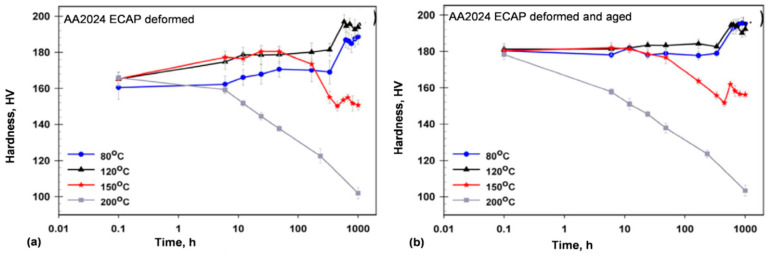
Thermal stability of nanocrystalline AA2024 alloy deformed by Equal Channel Angular Pressing (ECAP): (**a**) heated in as-deformed state; (**b**) heated after deformation and peak aging for 1 h at 190 °C [73].

Therefore, low thermal stability restricts the application expansion of nanocrystalline alloys. To improve their thermal stability, the mobility of grain boundaries should be reduced, for example, through alloying. The precipitation-hardened AA2024 (Al−4.2Cu−1.5Mg−0.6Mn−0.5Si) alloy, with nanocrystalline structure, generated through the single-pass Equal Channel Angular Pressing, preserved its properties at 120 °C for up to 1000 h [73]. As shown in Figure 19, after long-term heating at 80 and 120 °C, a secondary hardening took place, whereas at 150 °C, softening was accompanied by a slight secondary hardening. In contrast, at 200 °C fast softening occurred. As the major cause of the hardness loss, the increased coarsening rate of the equilibrium phase S (Al_2_CuMg) accompanied by dislocation annihilation were identified. Moreover, the dislocation-rich structure and Mg clusters, remaining from the S precipitate dissolution, eased the nucleation of Ω precipitates, which were responsible for the secondary hardening. The above transformation sequence differs from that described for the AA2024 commercial sheet [74].

As another example, the nanocrystalline structure of Al−Mg−Sc alloys, generated by Friction Stir Processing, was stabilized by the Al_3_(Sc,Zr) dispersoid phase up to 450 °C for 16 h [75]. In turn, in cold-worked Al–Fe–Si alloys, developed for electrical engineering purposes, the Mn−Ni−Cr fine dispersion phases were effective recrystallization barriers, preserving the microstructure up to 300 °C [76]. In another example, the nanocrystalline Al−10Fe−5Cr (wt.%) bulk alloy preserved the compressive strength of 450 MPa at 450 °C with such a high thermal stability being attributed to the formation of Fe and Cr containing phases with Al, such as Al_6_Fe, Al_13_Fe_4_, and Al_13_Cr_2_, in addition to the supersaturated solid solution of Cr and Fe in Al matrix [77].

The application of High Pressure Torsion to the Al−2.46Cu−1.48Mg−0.89Fe−0.92Ni (wt.%) alloy led to grain refinement to 200 nm, formation of high-angle grain boundaries and dynamic precipitation of Al_9_FeNi particles, with the strength preserved up to 225 °C [78]. It is of interest that 0.5 h annealing at around 225 °C reduced hardness to the level seen before deformation. Increasing the temperature up to 300 °C resulted in the same hardness of both deformed and non-deformed alloys. Similarly, high pressure torsion led to an improvement of tensile strength of the AA2198−T8 Al–Li alloy, associated mainly with grain refinement and dislocation strengthening [79]. However, aging of that alloy at 175 °C for 12 h caused a reduction in strength, indicating the low thermal stability. In contrast, the ultrafine grain AA8090 Al–Li alloy with an average grain size of 2 μm, obtained by Repetitive Corrugation and Straightening (RCS), was fairly stable during heating up to 300 °C [80].

Additions of Er to Al−Mg alloy powder improved its thermal stability through the combined effects of the solute/impurity drag and second-phase pinning, involving nanosize oxides, nitrides, and oxynitrides that impeded the grain boundary motion [47]. Small additions of the order of 0.1 wt.% Er were not effective and Al−Mg powders showed the abnormal grain growth at 180 °C. In contrast, the 0.5 wt.% Er addition improved thermal stability, maintaining the grain size of approximately 20 nm up to 400 °C. The controlled grain growth at higher temperatures resulted in an average grain size of 55 nm and a maximum observed grain size of ~200 nm after one hour of annealing at 500 °C.

## 6. Efforts to Increase the Upper Service Limit of High-temperature Grades

### 6.1. Development Drive for High-temperature Aluminum Alloys

**Figure 20:**
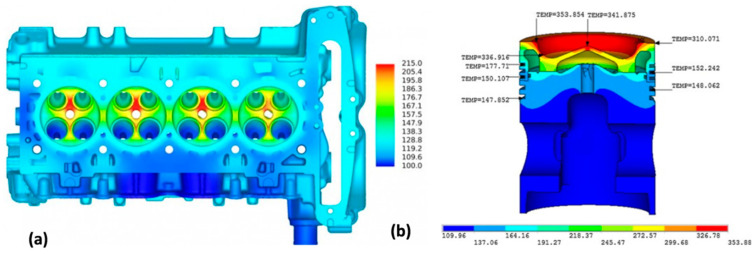
Computer simulation of temperature distribution within a combustion engine block with maximum temperature reaching 215 °C (**a**) [2,81] and thermal fields of a diesel engine piston with maximum temperature exceeding 350 °C (**b**) [82].

The vast application market and commercial opportunities create very strong drive for development of aluminum alloys with high-temperature capabilities. Despite of essential differences between the high-cost, high-end alloys for aerospace use and generally lower cost grades, required for large volume applications in ground transportation, both sectors fuel the critical research and development activities. The automotive interest is driven by new designs of combustion engines (both diesel and direct fuel injection gasoline engines), where an improvement in combustion efficiency requires the increased operating temperature up to 300 °C and internal pressure in and around the combustion chamber (Figure 20). Thus, aluminum alloys, capable of high-temperature strength and fatigue can increase performance and efficiency of combustion engine components through the lower weight and higher operating temperatures.

### 6.2. Concepts of Alloy Design for High-Temperature Service

A development of new Al alloys with high-temperature capabilities is not a trivial task and different directions are researched in academia and industry, including optimizing the conventional compositions and verifying the exotic solutions. During this search, an analogy is frequently made to nickel-based superalloys, which resist degradation of mechanical properties up to approximately 75% of their absolute melting temperature [83]. According to this concept, aluminum-based alloys should resist degradation up to 425 °C. The strengthening and thermal stability in Ni superalloys is achieved through ordered Ni_3_(Al,Ti) precipitates having the cubic L1_2_ structure, being isomorphous with the fcc Ni alloy γ matrix. By analogy with that, the high-temperature aluminum alloys must contain a high volume fraction of a dispersed phase, which has L1_2_ crystal structure and is thermodynamically stable at the intended service temperature. In this respect, trialuminide intermetallic compounds (Al_3_M) have a number of valuable features and are seen as perfect candidates in this role. The possible alloying elements are listed in Table A4, Appendix A.

### 6.3. Copper and Silver in Heat-Resistant Aluminum Alloys

As a potential structural material for use in aircraft structures operating at high temperatures, high strength aluminum alloys belonging to AA2XXX have been utilized for the past several decades. The alloys are commonly used for pistons and rotating aircraft parts due to their ability to work in higher temperature applications. During service at high temperature, stability of precipitates is still a major concern.

#### 6.3.1. Al−Cu−Mg

The heat-resistant wrought aluminum alloy, designated as AA2618 (2618A), has been widely used in aerospace since the 1950s with extensive research conducted on its thermal stability [84]. In this alloy, the properties and thermal stability are controlled by Cu and Mg contents with high Cu content in solid solution accelerating the precipitation coarsening [85]. For 3.1−3.7Cu and 1.2−2Mg, strengthening by S’ (Al_2_CuMg) yield stress of 465 MPa (T8) and good thermal stability up to 200 °C exist. For 4.8−5.4Ce and 0−0.4Mg, strengthening by θ’ (Al_2_Cu) yields stress of 380 MPa and good thermal stability up to 300 °C is valid. For 3.7−4.3Cu and 0.9−1.3Mg, strengthening by S’ and θ’ yields stress of 470 MPa but poor thermal stability of 150 °C was found (all values in wt.%.).

#### 6.3.2. Al−Cu−Mg−Ag

The Al−Cu−Mg system, with a high Cu−Mg ratio and a minor addition of Ag, is the base system for aerospace applications. Although there are differences between alloys from the Al−Cu−Mg−Ag family, the tensile strength decreases gradually with increasing the thermal exposure time at 200–250 °C. The combined addition of small concentrations of Ag and Mg to Al−Cu alloys promotes precipitation of Ω phase that forms as thin, hexagonally shaped plate-like precipitates on {111}α matrix planes. The uniform dispersion of this precipitate as well as θ’ plates on the {001} planes are considered to be the major contributor to alloy hardening. The thermal stability and coarsening resistance of microstructures dominated by Ω plates was found to coincide with relatively high levels of Ag and moderate Mg additions, with the latter limiting the competition for solute with S-phase precipitation [86].

The Al−Cu−Mg−Ag alloy maintained a tensile strength of 434 MPa after 1000 h at 150°C, being 86% of that of the peak-aged alloy [87]. After exposure for 1000 h at 200 °C the tensile strength decreased to 307 MPa. The improved alloy from this family, Al6.5Cu0.3Mg0.5Ag (wt.%) trademarked as KS2000, with high Cu/Mg ratio of 20, higher than in AA2618 Al1Fe2.5Cu1.5Mg1Ni (wt.%), was developed by the refinement of chemistry, heat treatment, and refinement of grain size through the optimization of forging conditions [9]. The alloy maintained the yield stress of 375 MPa after 100 h exposure at 150 °C, as compared to 330 MPa achieved by AA2618−T61.

The possibility of Al−Cu−Mg−Ag alloying with Zr and Sc led to nanoscale dispersoids of Al_3_(Sc,Zr) under solution treatment with no primary AlCuSc phase [88]. The dominant precipitation was still the Ω phase, formed on the {111}α planes and being susceptible to coarsening at 200 °C, thus determining the alloy thermal stability.

### 6.4. Rare Earth Metals in Aluminum Alloys

Rare earth metals have attracted increasing interest during aluminum alloying not only as minor additives, but also as their major ingredients. There are examples with an application of mischmetal and individual elements with positive effects, no effects [89], and detrimental influence on properties of aluminum alloys [90]. Rare earth elements such as La, Ce, Nd, and Y were tested with the Al−Si matrix [91]; Er with Al−Mg, Al−Zn−Mg, Al−Zn−Mg−Cu, Al−Li, and Al−Cu grades [92]; or Sm with Al−Si−Cu grades [93]. Among rare earths, cerium and scandium are of particular interest for aluminum alloying, as emphasized in [94,95,96].

#### 6.4.1. Exploring Scandium to Improve Thermal Stability of Al

Aluminum−scandium alloys have excellent mechanical properties at ambient and elevated temperatures due to the presence of coherent, nanoscale, L1_2_-ordered Al_3_Sc precipitates with a gain in tensile strength per atomic percent of scandium higher than for any other alloying element added to aluminum. The key effects of scandium include (i) grain refinement during casting or welding, (ii) precipitation hardening from Al_3_Sc particles, and (iii) grain structure control from the Al_3_Sc phase. An addition of scandium in a combination with zirconium is particularly effective, which is linked to the core/shell structure of the Al_3_(Sc,Zr) dispersoids [94].

Due to unfavorable diffusivity of Sc in Al, the thermal stability of alloys is rather moderate. For Al−5Mg−0.3Sc (wt.%) alloys, heating at 300 to 450 °C resulted in increasing the Al_3_Sc coarsening rate by an order of magnitude for every 50 °C [97]. However, Al_3_Sc precipitates exhibited good coherency with the aluminum matrix, even after being annealed at 300–450 °C for 168 h. Coarsening resistance of Al_3_Sc precipitates was described in [98,99]. Unfortunately, the prohibitive cost barrier accompanies an extraordinary improvement of aluminum properties by micro-additions of scandium.

#### 6.4.2. Influence of Other Rare Earths

Similarly to Sc, the four heaviest rare earth elements, Er, Tm, Yb, and Lu, develop an Al–Al_3_RE eutectic reaction, with Al_3_RE having the stable L1_2_ crystal structure. The available data refer to experimental alloys, synthesized in small volumes in a laboratory environment. The binary aluminum alloys with 0.03–0.06 at.% Yb or Er, after aging produced the coherent, nanosized Al_3_RE precipitates [100]. Based on microhardness data from isochronal aging experiment, the Er precipitation started at 150 °C and an aging peak occurred at 275 °C for Al–0.03Er, while for Al–0.03Yb precipitation of Yb started at 100 °C and an aging peak occurred at 250 °C. The significantly lower temperatures obtained for Er and Yb alloying as compared to the Al–0.12wt.% Sc alloy is explained through larger diffusivities in Al as compared to Sc. This claim, however, is not supported by diffusivity data in Table A3, Appendix A.

There are many examples of using the minor additions of mischmetal or Ce with both the experimental and commercial alloys [96]. In addition to some improvements in thermal stability, there is also evidence of no beneficial effect of Ce additions. For example, after alloying of the A205/TiB_2_ composite with 0.2–1.5 wt.% Ce, no influence on thermal stability of the Ω phase, tested at 230 °C for up to 100 h, was recorded [101]. It is claimed that cerium was located in the CeTi_2_Al_20_ phase and poisoned the matrix grain refinement by nucleating around the TiB_2_ particles.

### 6.5. Features Specific for Cast Alloys

In this report, no clear separation is made between wrought and cast alloys. Based on the chemical composition and manufacturing route provided, the alloy can easily be classified to which group it belongs. In this section, some features specific for development of cast alloys with high-temperature capabilities are described.

There are many benefits of using cast alloys to manufacture components for engineering applications; in contrast to the multistage manufacturing cycle, typical for wrought products, through casting, e.g., through modern high pressure die casting (HPDC). However, there are also disadvantages, with the most important one for structural applications being the component poor integrity. In addition to improving casting technology, including mold (die) design and selection of process parameters to control flow of molten metal during die filling, post-casting treatments may be used. One of them is hot isostatic pressing (HIP), aimed at eliminating pores and bonding pore surfaces together. In some applications, the pneumatic isostatic forging was an effective technology for improving mechanical properties in aluminum castings through healing defects in castings and controlling the microstructure [102]. In cast alloys, segregation, being also their inherent feature, may have detrimental effect on alloy thermal stability.

**Figure 21:**
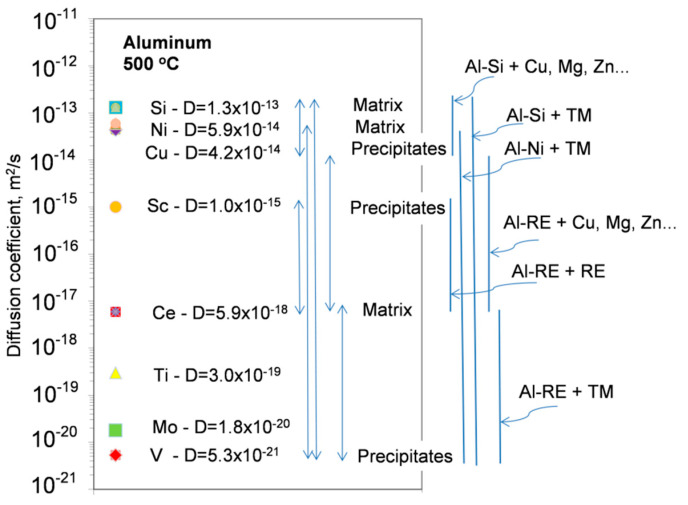
A development strategy of cast Al alloys with high temperature stability through combining of appropriate matrix (Al−Si, Al−Ni, Al−RE) and strengthening compounds (conventional elements, RE, TM). Strengthening can be effective after casting or/and after post-casting heat treatment. Diffusion coefficients of alloying elements in aluminum at 500 °C were calculated based on data in Table A1, Table A2 and Table A3 in Appendix A. RE—rare earths; TM—transition metals.

The graph in Figure 21 shows a strategy for selection of alloy matrix and alloying elements, when considering differences in their diffusivities in aluminum.

Four development criteria for cast, precipitation-strengthened aluminum alloys for high temperature applications were formulated: (i) solid state precipitation of Al_3_M with L1_2_ crystal structure; (ii) appropriate shape of solvus line to facilitate precipitation; (iii) low diffusivity in of M in Al; and (iv) solid−liquid partition near unity to minimize segregation [83]. As in cast aluminum alloys eutectic is the essential component that provides the necessary fluidity in liquid state, to achieve a high thermal stability, the solidified eutectic structure should remain stable at service temperatures. The properties of potential alloying candidates are listed in Appendix A, Table A4.

#### 6.5.1. Improving Thermal Stability of Al−Si Matrix with Transition Metals

The Al−Si cast alloys are the most commonly used, representing up to 90% of the total aluminum cast parts, produced for the automotive industry. The operating temperature of existing Al−Si casting grades, for example, hypoeutectic Al−Si−Cu alloy A380 and Al−Si−Mg, typically below 180 °C, is becoming insufficient for modern designs of automotive engines.

Based on the diffusion coefficient criterion, the high temperature mechanical performance of traditional Al–Si alloys can be enhanced by alloying with transition metals, leading to formation of thermally stable and coarsening-resistant precipitates. Transition metals used in aluminum alloys, such as Mn, Fe, Co, Ni, Cu, Ag, and Zn, form equilibrium phase diagrams of eutectic type, when Cr, Mo, Ti, Zr, and V form diagrams of peritectic type (Appendix A, Table A4). They exhibit low solid state solubility in aluminum with limits mostly from a fraction of 1 to less than 2 wt.%, which sharply decreases with temperature. Moreover, the high solidus temperature, approaching melting point of Al, leads to narrow solidification range of Al−TM solid solutions. Therefore, their tri-aluminides with the general chemical formula Al_3_TM, formed within the aluminum matrix during artificial aging, have high thermal stability. There are data suggesting benefits of using combinations of two or more transition metals, additions of which widen the aluminum solid solution range [103].

##### Al−Si-Based Alloys Strengthened with Zr, V, and Ti

In aluminum alloys, individually added V forms the Al_3_V dispersoids with an Ll_2_ structure in the metastable condition and a D0_22_ structure in the equilibrium condition. The purpose of Zr additions is to form fine precipitates of intermetallic particles that inhibit recrystallization. It also forms a solid solution of Zr in Al. The major role of Ti is to refine the grain of aluminum matrix. In case of cast alloys, the Al_3_TM intermetallics, acting as nuclei during solidification, may lead to grain refinement. At the same time, the primary Al_3_Ti and Al_3_Zr compounds that form during solidification negatively affect the ingot ductility. According to ternary Al–TM1–TM2 phase diagrams, some combinations of transition metals do not decrease their joint solid state solubility in aluminum and widen the range of existence of the aluminum solid solution [104]. Such combinations are also beneficial to suppress the precipitation of primary intermetallics during solidification, being detrimental to the alloy performance.

**Figure 22:**
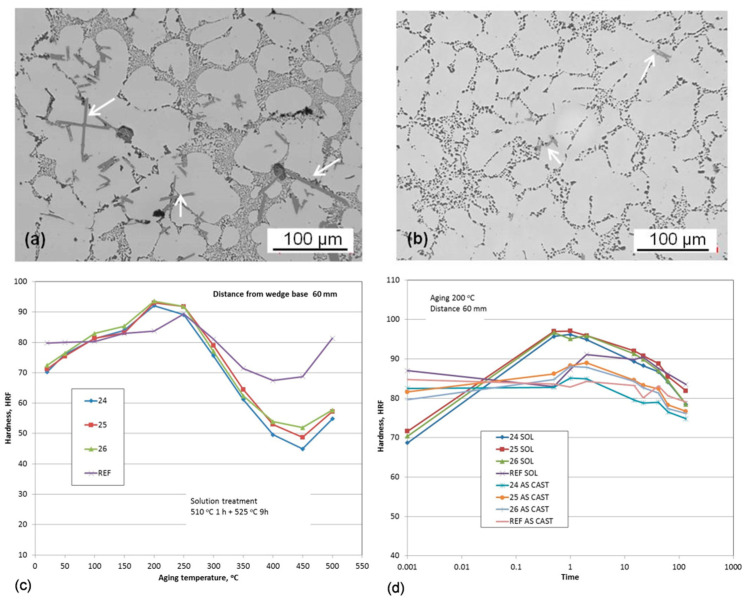
Microstructure of the Al7Si1Cu0.5Mg alloy modified with 0.47 wt.% Zr, 0.21 wt.% Ti, 0.30 wt.% V in as-cast condition (**a**) and after T6 heat treatment (solutionizing 510 °C: 0.5 h + 525 °C, 4.5 h water quench; aging 200 °C, 1 h air cooling). Arrows indicate coarse phases containing transition metals Ti−Zr−V (**b**). Effect of aging temperature (**c**) and time (**d**) on hardness for various additions of transition metals. Figure legend: (24) 0.14 wt.% Zr, 0.14 wt.% Ti, 0.22 wt.% V; (25) 0.42 wt.% Zr, 0.18 wt.% Ti, 0.17 wt.% V; (26) 0.47 wt.% Zr, 0.21 wt.% Ti, 0.30 wt.% V; (REF) A380 alloy.

A combination of Zr, V, and Ti additions with individual contents from 0.2 to 0.5 wt.% was explored to modify the hypoeutectic Al7Si1Cu0.5Mg (wt.%) cast alloy [105,106]. The objective was to modify its phase composition and aging characteristics, and create intermetallic phases with sufficiently high thermal stability to improve the alloy performance in potential applications for automotive engine blocks and cylinder heads. As shown in Figure 22, the results of isochronal aging, performed at temperatures up to 500 °C, revealed that the peak aging hardness occurred at approximately 200 °C. An assessment of the role of time shows that at 200 °C, solution-treated alloys with Zr−V−Ti increased hardness, reaching a peak value after roughly 1 h exposure; then hardness reduction was observed.

Micro-additions of Ti, V, and Zr to the Al–7Si–1Cu–0.5Mg (wt.%) cast alloy led to the formation of (AlSi)_x_(TiVZr) phases with increased stability at high temperatures, thus positively affecting the alloy strength. The improvement in tensile and compressive strength was preserved up to 200 °C with more positive effect seen for the T6 state. The hardness curves show that the potential alloying benefits from transition metals Zr−V−Ti are not fully utilized. Being effective, they should exhibit much higher hardness advantage over temperature and time than the A380 alloy with Cu−Si containing compounds.

Hardness tests do not reveal the entire picture of alloying with Ti, Zr, and V and benefits in improving thermal stability are shown through the higher creep rapture strength and much longer fatigue life than that of the A380 alloy, currently used by the automotive industry [107].

##### Al−Si Alloys with Micro-Additions of Zr, V, Ti and Cr

According to literature data [108], the presence of Cr in Al−Si cast alloys changes the morphology of the β−AlFeSi phase from the needle-like to a fishbone-like α−Al(Fe,Cr)Si phase, leading to an improvement of ultimate strength. Unknown remains the synergy of simultaneous presence of Ti, Zr, V, and Cr. Thus, the objective of Cr additions was to verify how the Cr−Ti−V−Zr-rich phases influence the alloy properties at high temperatures [109]. To achieve this, the base Al7Si1Cu0.5Mg (wt.%) alloy was modified with 0.39 wt.% Ti, 0.25 wt.% Zr, 0.39 wt.% V, and 0.47 wt.% Cr. The alloy base used was the same as in previous section, thus allowing direct comparison of results.

A presence of additional transition metal Cr contributed to noticeable microstructural differences to those described for Zr−V−Ti additions. The Cr-containing phases included Al18.5Si7.3Cr2.6V (wt.%) with needle shape and Al7.9Si8.5Cr6.8V4.1Ti (wt.%) having hexagonal pipe shape, most likely falling into the general formula as (AlSi)_3_(CrV) and (AlSi)_3_(CrVTi), respectively.

**Figure 23:**
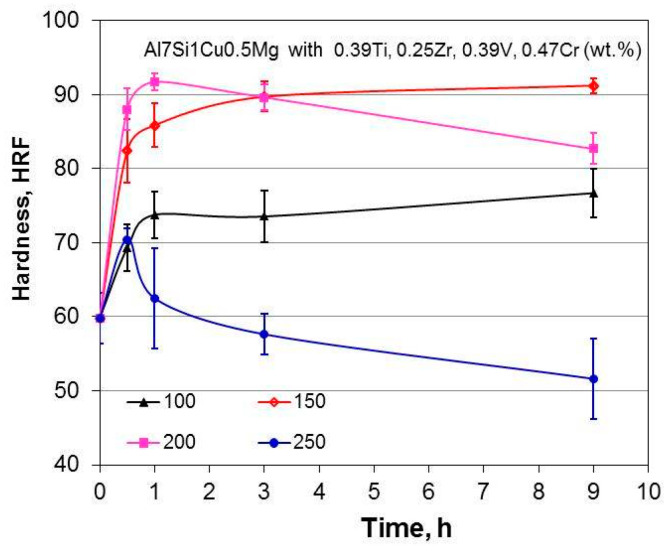
Hardness changes during aging of the Al7Si1Cu0.5Mg alloy modified with 0.39 wt.% Ti, 0.25 wt.% Zr, 0.39 wt.% V, and 0.47 wt.% Cr. Solution treatment included heating at 510 °C for 0.5 h followed by 4.5 h at 525 °C and water quench [109].

The hardness changes versus aging time for different aging temperatures of the Al7Si1Cu0.5Mg alloy, modified with Ti−Zr−V−Cr, are shown in Figure 23. The solution treatment reduced the alloy hardness and, according to heat treatment observations, it looked more sensitive to the aging temperature rather than to the aging time. Based on comparison with alloys described in the previous section, it may be concluded that additions of transition metal Cr to Zr, V, and Ti did not substantially change the thermal stability of the Al7Si1Cu0.5Mg base. It appears again that all transition metals were concentrated in coarse primary compounds, having low effectiveness in as-cast conditions. Also solution annealing was not able to dissolve them completely and drastically change their effectiveness after aging.

##### Al−Si Alloys with Micro-Additions of Mo and Mn

The presence of molybdenum in Al alloys leads to nanoscale dispersoid formation, which contributes to improved high-temperature strength and creep resistance. Micro-additions of Mo to commercial Al−Si alloys slightly improve strength at room temperature, but substantially improve the alloy thermal stability with preserving properties up to 250 °C.

Another transition metal manganese has very low diffusion coefficient in aluminum and form high density of micron-size primary Al_6_Mn, Al_6_(Mn,Fe), and Al(Mn,Fe)Si precipitates in alloys containing Si and Fe. A combination of Mn with Mo provides strengthening through Al(Mn,Mo)Si precipitates and looks promising in terms of Al thermal stability improvement. As reported in [110], Mo additions to the Al7Si0.5Cu0.3Mg alloy (wt.%) led to formation of α−Al(Fe,Mo)Si and α−Al(Fe,Mn,Mo)Si nanoprecipitates with and without Mn.

**Figure 24:**
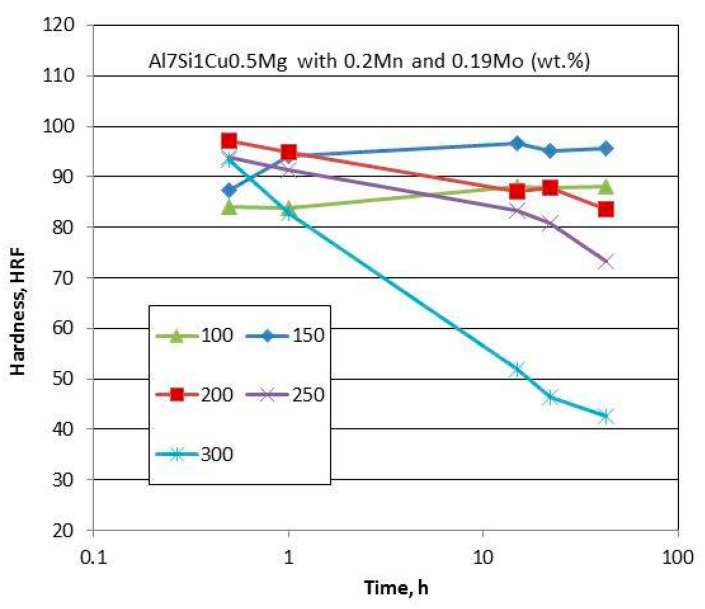
Hardness changes during aging of the Al7Si1Cu0.5Mg alloy modified with addition of 0.20 wt.% Mn and 0.19 wt.% Mo. Solution treatment included heating at 510 °C for 0.5 h followed by 4.5 h at 525 °C and water quench [111].

In the as-cast state, the Al7Si1Cu0.5Mg (wt.%) alloy with micro-additions of 0.20 wt.% Mn and 0.19 wt.% Mo was composed of dendritic α−Al; fibrous-like eutectic Si; and Cu-, Mg-, and Fe-based phases with Mo and Mn additions being present in the α−Al17Fe3.2Mn0.8Si2, Al65Cu20Fe6Mn9, Al13Fe8Mn33Mo, and Al0.78Fe4.8Mn0.27Mo4.15Si2 phases [111]. An example of the influence of artificial aging on alloy hardness is shown in Figure 24. The hardness test revealed that the Al7Si1Cu0.5Mg alloy with Mo and Mn was able to retain its hardness for maximum aging time of 75 h up to 200 °C. There was some hardness reduction for temperatures of 200 and 250 °C but the evident softening was observed after exposures at temperature as high as at 300 °C. The overall conclusion is that the Al−Si−Cu−Mg alloy with Mo−Mn combined additions showed better hardness retention than achieved for the same base, modified with Zr, Ti, V, and Cr.

#### 6.5.2. A Search for Novel Matrix of Cast Aluminum Alloys

An alloy designed for casting should contain the eutectic component, providing sufficient fluidity in molten state and strengthening dispersoids. The latter ones may form directly during solidification or they can precipitate as a result of a subsequent heat treatment. In practice, both forms of strengthening compounds often coexist. Therefore, thermal stability of the solidified eutectic and strengthening phases affects the alloy performance at increased temperatures.

##### Eutectic Based on Al−Ni System

The presently used Al−Si eutectic with low melting range limits the stability improvement [35]. To overcome this barrier, the Al−Al_3_Ni eutectic was proposed [112,113,114] with better thermal stability, attributed, according to the work in [112], to the existence of a thin coherent layer of α−Al surrounding each Al_3_Ni fiber. However, among other issues of Ni, the fluidity of the Al−Al_3_Ni eutectic, tested in the Al−6Ni−4Mn (wt.%) alloy, was not as high as that of typical casting alloys, e.g., A390. The Al−Al_3_Ni eutectic matrix is also weak, so additional alloying is required to improve mechanical properties and to preserve the fiber thermal stability.

In an effort to improve strength, Sc was used in [115,116]. The eutectic in the Al−6wt.%Ni alloy with Al_3_Ni microfiber morphology maintained its hardness at 300 °C for up to 672 h. Additions of 0.4 wt.% Sc did not change the morphology of the Al_3_Ni microfibers and did not change the alloy thermal stability; the major effect of Sc was expressed through increased the alloy strength. The strengthening contributions of the Al_3_Ni and Al_3_Sc phases at ambient temperature are cumulative in the ternary alloys. The hardness curve of Al−6Ni−0.4Sc (wt.%) at 300 °C was described via a superposition of the curves of Al−0.4Sc and Al−6Ni alloys, over the full aging time of 0–672 h. In another research, a new generation of heat-resistant aluminum alloys, based on Ni-containing eutectic, the Al–Ni–Mn–Fe–Si–Zr system, strengthened by the Al_3_Zr (L1_2_) nanoparticles, was developed [117]. The presence of Si widened the crystallization range, increasing the tendency of the alloy to form hot cracks during casting but having high thermal stability.

##### Eutectic Based on the Al−Ce System

A possibility of further improvement in thermal stability of aluminum alloys is anticipated through a substitution of nickel with a rare earth metal cerium, having the diffusion coefficient in aluminum lower by approximately four orders of magnitude than that for nickel [96]. At present, there is still no convincing evidence, supporting the Al−Ce system in this role.

**Figure 25:**
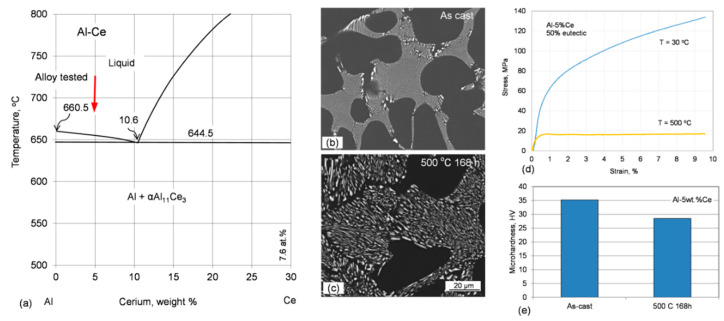
Thermal stability of Al−5 wt.% Ce binary alloy: (**a**) portion of Al−Ce binary diagram showing location of commercially important eutectic [124]; (**b**) eutectic morphology after casting; (**c**) eutectic morphology after heating for 168 h at 500 °C; (d) stress versus engineering strain plot for alloy compression at room and at 500 °C; (**e**) hardness after casting and heating for 168 h at 500 °C.

The idea of using cerium for aluminum alloying with contents, reaching eutectic compositions, was introduced in 1912 [118], followed by research in 1920s [119,120,121] with a summary of early efforts given in [122]. Historically, exploring Ce as the major alloying element of Al, where it forms the Al_11_Ce_3_ eutectic phase with melting point of 1251 °C, aimed at increasing the thermal stability of alloys [96]. According to recent statements [123], alloying with Ce helped retaining the mechanical properties of Al alloys at higher temperatures than that seen in Ce−free Al grades, with Al−Ce showing complete hardness recovery at room temperature after exposures to temperatures as high as 500 °C for 1000 h. There is research, however, documenting rather detrimental influence of heat on Al−Ce system. As shown in Figure 25, conventionally cast Al−5wt.%Ce binary alloy exhibited drastic reduction in strength at 500 °C and 20% hardness reduction after exposure for 150 h at 500 °C.

**Figure 26:**
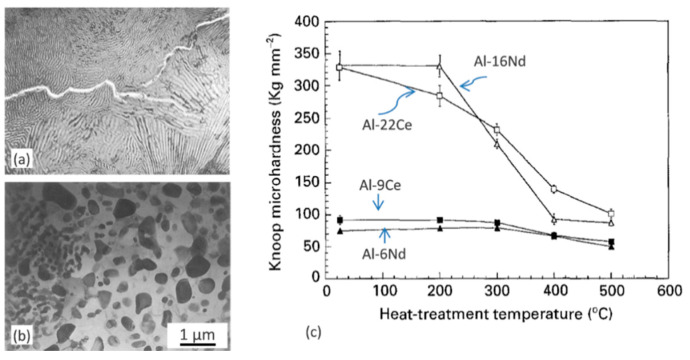
Thermal stability of rapidly solidified Al−Ce and Al−Nd alloys: (**a**) Al−22Ce as spun cast; (**b**) Al−22Ce after 2 h at 500 °C, TEM images; (**c**) microhardness after heating at temperatures from 200 to 500 °C for 2 h (alloy compositions are in wt.%) [125].

The hardness reduction was accompanied by a substantial change in the eutectic morphology with visible lamellae modifications. Moreover, the rapidly solidified Al−Ce and Al−Nd alloys, with Ce and Nd contents up to 22 wt.%, experienced a substantial reduction in hardness, accompanied by morphological changes with a disappearance of lamellae after exceeding 300 °C (Figure 26) [125]. Another study, using the spun cast Al−8 wt.% Ce and Al−20 wt.% Ce alloys essentially confirmed the above observations. Although Al−Ce alloys preserved the initial hardness, when annealed at 200 or 300 °C, they experienced sharp softening after annealing at 400 or 500 °C for 0.5 h [126].

## 7. Alloy Stability Improvement through Processing Techniques

In addition to optimizing the alloy chemical composition, to improve thermal stability, parallel efforts are explored through alloy processing, such as rapid solidification, powder metallurgy, and additive manufacturing, engineering alloys in a liquid state, and post-casting treatments. They represent either modification of conventional or testing novel manufacturing routes or, as in the case of liquid metal engineering, an extra step within the conventional manufacturing sequence. Each technique exerts its unique impact on microstructural constituents, affecting the alloy performance at increased temperatures.

### 7.1. Liquid Metal Engineering

Liquid metal engineering refers to a variety of physical and/or chemical treatments of molten metals aimed at influencing their solidification characteristics [127]. It is generally accepted that exploring the synergy of melt chemistry and physical treatments, achieved through liquid metal engineering, allows creating the optimum conditions for nucleation and growth during solidification, positively affecting the quality of alloys.

#### 7.1.1. Refining Microstructure of Alloys Containing Transition Metals

**Figure 27:**
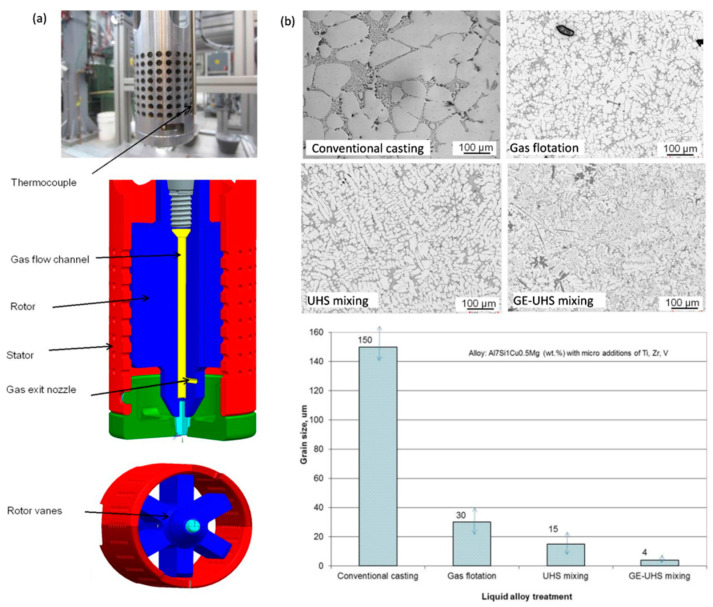
Application of Gas−Enhanced Ultrahigh Shear Mixing for refining microstructure of Al7Si1Cu0.5Mg alloy with 0.47 wt.% Zr, 0.21 wt.% Ti, 0.30 wt.% V: (**a**) mixing device; (**b**) microstructure after different treatments in liquid state with resultant grain size shown on histograms [129].

The detrimental feature of aluminum alloys modified with transition metals, having much higher melting points than aluminum, is that the intermetallic compounds that control thermal stability are generally coarse and therefore ineffective. Thus, an opportunity to refine the coarse compounds, using liquid metal treatment, offers a number of benefits through reducing the overheating temperature, required during melting, shortening the holding time in a molten state and shortening the holding times during post-casting heat treatment, or in some cases, eliminate a need for heat treatment altogether. A new mixing technology that explores an integration of gas injection into the shear zone with ultrahigh shear mixing, called Gas-enhanced Ultrahigh Shear Mixing (GE−UHS), allows refining the microstructure through affecting the alloy solidification mechanism (Figure 27) [128].

An application of the GE−UHS technology to molten aluminum alloys shows that injecting gas into a shear zone of the rotor/stator apparatus drastically magnified the alloy structural refinement, which substantially exceeded the individual effects, caused by gas flotation and ultrahigh shearing. For an experimental Al−7Si−1Cu−0.5Mg (wt.%) alloy with microquantities (0.1 to 0.5 wt.%) of V−Ti−Zr, in addition to matrix grain size reduction by almost two orders of magnitude, the complex intermetallic compounds (AlSi)_x_(TiVZr) with D0_22_/D0_23_ tetragonal crystal structure were refined [129]. Those compounds with transition metals are crucial for thermal stability but remain inherently coarse in conventional castings.

#### 7.1.2. Morphology Change of the Eutectic Phase in Alloys with Additions of Rare Earths

For some alloys, liquid metal engineering may be the only option to refine the strategic strengthening compounds. An example is the Al−Ce alloy, where due to negligible solid state solubility of Ce in Al, generation of strengthening precipitates through heat treatment is impossible. The key component of Al−Ce binary alloys, which controls their thermal stability, is the Al−Al_11_Ce_3_ eutectic. However, the Al_11_Ce_3_ eutectic phase with lamellar morphology provides limited strengthening to the alloy [124].

According to recent experiments, the eutectic morphology can be transformed from lamellar to more effective fiber-like, using an agitation of a molten alloy before solidification with a permanent magnet [130]. Modeling of the lamellar-to-fiber transition within the Jackson–Hunt framework, followed by experiments, using the Al−5 wt.% Ce alloy, led to the fiber structure with improved mechanical properties. The alloy processing through permanent magnet stirring at 630 °C exhibited increased mechanical strength retention and better performance than the other commercial aluminum heat-resistant alloys. Although, there is some ambiguity, regarding the alloy treatment temperature and its location in regards to the alloy liquidus, this example demonstrates that liquid metal engineering offers a new route to influencing the solidification morphology with a considerable advantage over directional solidification and laser additive manufacturing.

### 7.2. Rapid Solidification

Rapid solidification technology is explored for decades with aluminum alloys, showing many advantages over conventional ingot casting. It improves the elevated temperature performance of aluminum alloys through high supersaturations of elements in the matrix. Several experimental materials with transition metals Fe and Cr, produced through this route, have promising creep properties up to 350 °C [131].

The rapid solidification was used to manufacture aluminum alloys for high temperature applications, including systems Al–Fe–Ce, Al–Fe–Cr–(TM), Al–Cr–Zr(Mn), and Al–Fe–V(Mo)–Si [132]. The best examples of green products show strength of 550–600 MPa at room temperature and at least 200–250 MPa at 300 °C. The strength of Al–Fe–Cr–(TM) alloys with a high volume content of quasicrystals is approximately 100 MPa higher at 20 and 300 °C, while their elongation is 50 to 67% lower than typically seen in other aluminum alloys. In particular, in the Al−8.8Fe−3.7Ce (wt.%) alloy, processed through arc melting and rapid solidification and followed by extrusion, led to formation of metastable phases in addition to equilibrium structures Al_6_Fe, Al_10_Fe_2_Ce, and Al_20_Fe_5_Ce [133]. The Al_20_Fe_5_Ce phase was a decagonal quasicrystal while the Al_10_Fe_2_Ce phase was determined to have an orthorhombic crystal structure belonging to space group C*mmm*, C*mm*2, or C222.

### 7.3. Mechanical Alloying—Alloy Consolidation from Fine Powders

Mechanical alloying is widely used to produce nanostructured aluminum alloys for high-temperature applications, including compositions with transition metals, where having the fine and homogenous microstructure is difficult to obtain after conventional casting. The process is generally implemented through a combination of gas atomization, ball milling, and hot pressing. Examples of alloys include both standard and unique compositions such as Al86Ni7Y4.5Co1La1.5 (at. %) [134], 70.0Al−22 Fe−8.0Ti (wt.%), 69.0Al−22 Fe−8.0Ti (wt.%) with 1.0 nano−Y_2_O_3_, and 69.0Al−22 Fe−8.0Ti (wt.%) with 1 wt.% nano−TiO_2_ [135].

**Figure 28:**
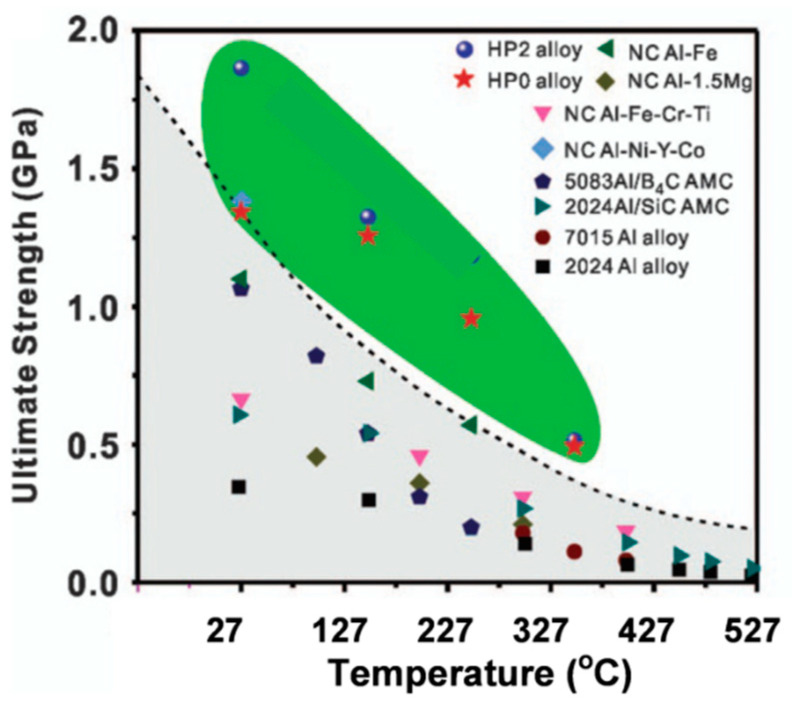
Thermal stability of the Al84Ni7Gd6Co3 (at.%) alloy, produced by a combination of gasification, ball milling and hot pressing as compared to other alloys taken from the literature. HP0—hot pressing at 640 MPa and T_HP_ = 500 °C using gas-atomized powders; HP2—powders milled for 50 h (HP1) and 100 h [25].

The powder metallurgy with devitrification and consolidation of amorphous/crystalline powders was also used to manufacture Al84Ni7Gd6Co3 (at.%) alloys with a unique hybrid microstructure, composed of isolated nanoscale fcc−Al grains and intermetallic compounds [25]. The high strength at both room and high temperatures is attributed mainly to the composite structure and the effect of confinement between the nanosized Al and intermetallic phases. As a result of the confining effect, the premature brittle fracture of the intermetallics and the nanocrystalline Al could effectively be suppressed, thus offering the possibility to deform plastically and to exhibit intrinsic strength rather than the flaw-controlled strength. An example of properties of the Al84Ni7Gd6Co3 (at.%) alloy are shown in Figure 28.

The combined mechanical alloying and powder consolidation was also found effective during manufacturing of the Al(Mg)−NiO composites with very fine grain matrix [136]. During processing, transformation of NiO particles into thermodynamically stable Al_3_Ni and MgAl_2_O_4_ compounds in the Al(Mg)−NiO composite extruded at 400 °C is responsible for the flow stress decrease, observed for a wide range of deformation temperatures. Therefore, the effect of preliminary annealing at 600 °C on the flow stress versus deformation temperature characteristics was very limited, because the chemical reaction occurred during the hot extrusion of the composite, i.e., before composite annealing.

### 7.4. Additive Manufacturing

Additive manufacturing (3D printing) covers a variety of computer-controlled processes, where a material is deposited layer by layer and solidifies to create a three-dimensional object. Selective laser melting and electron beam melting represent the major technologies of additive manufacturing. The key feature of selective laser melting, where manufacturing is conducted by repeated melting and solidification of a metal powder by laser, is its cooling rate, being much faster than that experienced during conventional casting [137].

In addition to refining structure due to a rapid solidification, multipass laser additive manufacturing can trigger precipitation hardening, thus replacing heat treatment. Conventional processing involves controlled ageing, during which the ordered and coherent Al_3_Sc precipitates form from a Sc-supersaturated solid solution. In [138], the intrinsic heat treatment that explores the deposition energy was used to trigger in situ precipitation of Al_3_Sc during laser additive manufacturing. As the intrinsic heat treatment causes precipitates coarsening, thereby reducing their strengthening effect, the alternative solidification conditions were implemented to exploit the intrinsic heat treatment to form the Zr-rich shell around the Al_3_Sc precipitates.

**Figure 29:**
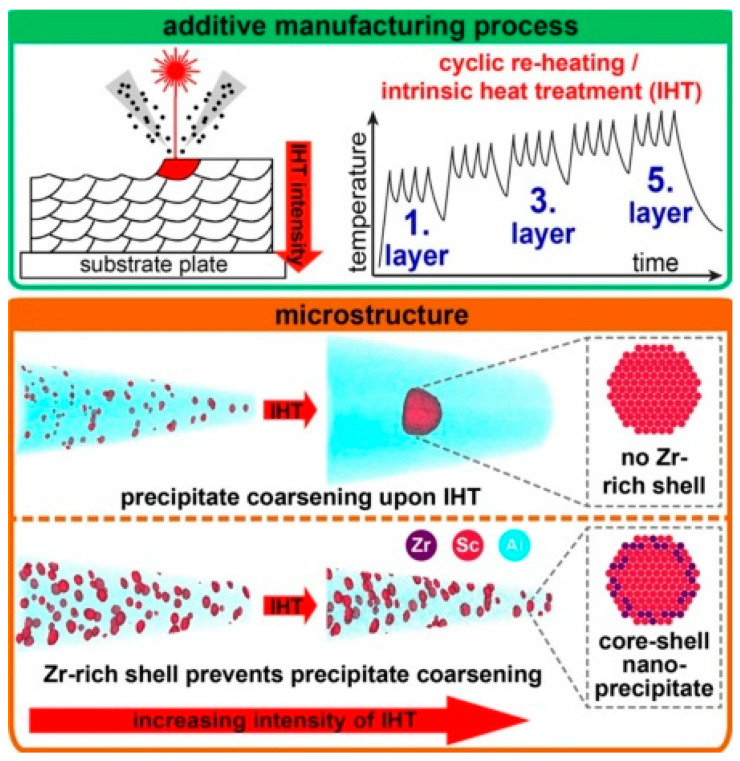
Control of thermally stable core−shell nanoprecipitates in additively manufactured Al−Sc−Zr alloys [138].

As shown in Figure 29, a presence of the Zr shell prevents the precipitate coarsening. This approach is applicable to a wide range of precipitation-hardened alloys, used in laser additive manufacturing.

In general, laser-printed metals do not tend to match the mechanical properties and thermal stability of conventionally manufactured alloys. However, laser additive manufacturing can also produce high-performance and near-net shape parts of aluminum matrix composite with higher specific strength, better wear resistance, and more outstanding physical properties than aluminum alloys, which are widely used in automotive and aerospace fields [139]. It should be kept in mind that a single-pass laser melting creates microstructural effects similar to those achieved during rapid solidification.

Laser melting overcomes a challenge of manufacturing the aluminum matrix nano−composites with a high density of dispersed nanoparticles. The laser printed aluminum nano composites with a thickness of around 300 μm, reinforced with 35 vol.% TiC reached a yield stress of up to 1000 MPa, elongation over 10%, and Young’s modulus of approximately 200 GPa [140].

**Figure 30:**
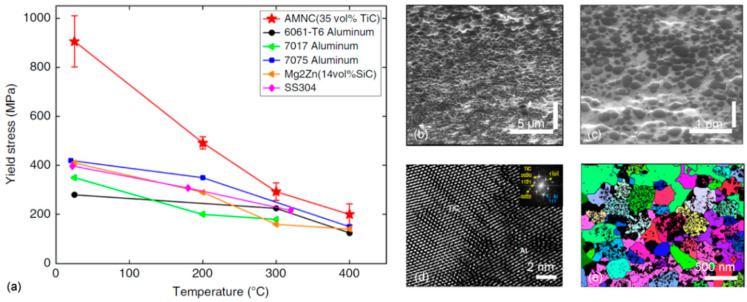
Mechanical behavior and microstructure of laser-deposited Al−matrix nanocomposites (AMNC) at elevated temperature: (**a**) yield strength of nanocomposite (35 vol.% TiC) at 25, 200, and 400 °C in comparison with other materials; (**b**,**c**) SEM images showing TiC nanoparticles uniformly distributed in matrix; (**d**) FFT-filtered high-resolution TEM image showing good bonding between TiC nanoparticle and Al; (**e**) grain map showing orientation distribution [140].

As shown in Figure 30, hardness of the composite over wide temperature range exceeds values obtained for other metallic materials listed there. An improvement in the composite performance is attributed to the high density of uniformly dispersed nanoparticles, strong interfacial bonding between particles and matrix, and fine grain sizes of ~330 nm. Similar benefits of selective laser melting were recorded after deposition of AlSi10Mg (wt.%) composites with additions of TiB_2_ reinforcement [141].

**Figure 31:**
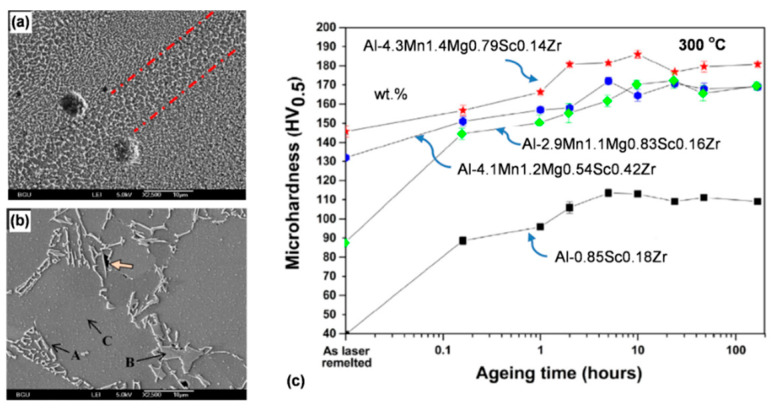
Refinement of microstructure of AlSi10Mg alloy after selective laser melting (**a**) as compared to the conventionally cast state (**b**), A—Al−Si eutectic, B—Si dispersion, C—Fe-containing intermetallics [143]; (**c**) aging responses of laser remelted Al−Mn−Sc and Al−Sc−Zr alloys at 300 °C [142].

To better exploit the advantages of additive manufacturing and optimize the functionality of the customized components, alloys specifically developed for this manufacturing route are required. At present, the majority of additive manufacturing of aluminum-based alloys involves commercial grades such as AlSi7Mg, AlSi12, and AlSi10Mg (wt.%), designed for conventional casting. An attempt is shown in Figure 31, where novel Al−Mn−Sc alloys were evaluated by selective laser melting [142]. Due to formation of the primary Al_3_(Sc,Zr) particles at the molten pool boundaries, the Al−Mn−Sc alloys developed a fine columnar-equiaxed bimodal grain structure with high thermal stability. Additions of transition metal Mn, forming Al_6_Mn likely through the solute rejection from the solidification front and nucleation at the grain and subgrain boundaries or dislocation walls, contributed to thermal stability improvement.

The combination of processing through selective laser melting and alloying with Fe improved the high-temperature strength and ductility of Al−11.6Si−0.97Cu−0.96Mg−1Ni (wt.%) alloys [144]. According to microstructural observations the improvement was caused by the dispersion of fine rod-shaped Fe−Si−Ni particles, which replaced the cell-like structure of eutectic Si, typical for the conventionally cast state. Selective laser melting was also found effective to fabricate components from the heat-resistant Al–8.5Fe–1.3V–1.7Si (wt.%) aluminum alloy with a microhardness of 246 HV0.1 [145]. The nanosize spheroidal Al_12_(Fe,V)_3_Si particles, homogeneously distributed in the Al matrix, were developed in the heat affected zone, while the rectangle-like Al_m_Fe phase with m = 4.0–4.4 and 100–500 nm in size was formed in the border re-melting zone.

In addition to experimental alloys, there are also commercial ones, designed for additive manufacturing. An example is the second-generation aluminum−magnesium−scandium (Al−Mg−Sc) alloy, referred to as Scalmalloy^®^, developed by Airbus research center as a high-strength lightweight alloy for selective laser melting [146]. The alloy with a specific weight of 2.67 g/cm^3^ offers tensile strength of 520 MPa with elongation of 13% and the microstructure stable up to 250 ° C.

## 8. Thermal Stability and Exposure to Accidental Fire

**Figure 32:**
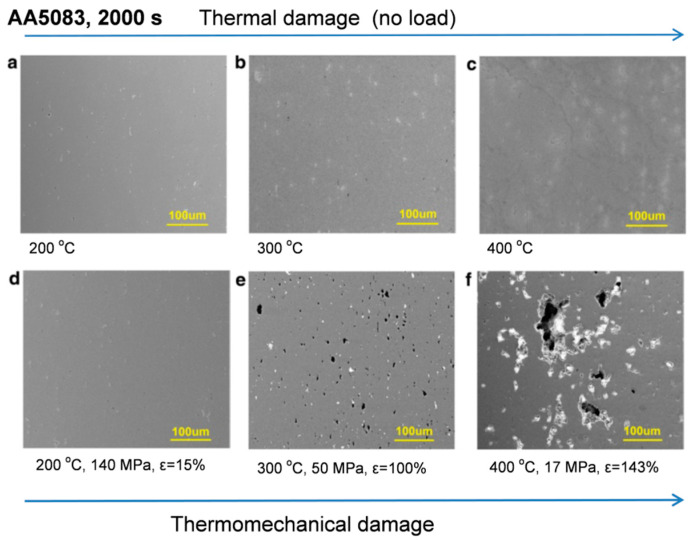
Comparison of (**a**–**c**) thermally and (**d**–**f**) thermomechanically damaged 5083−H116 alloy exposed for 2000 s. The shown conditions are (a) 200 °C, (b) 300 °C, (c) 400 °C, (d) 200 °C, 140 MPa, ε = 15%, (e) 300 °C, 50 MPa, ε = 100% and (f) 400 °C, 17 MPa, ε = 143%. The rolling/loading direction for all micrographs is along the long axis of the page [10].

In case of accidental fire hazards a metal might be exposed to enormously high temperatures. Due to relatively low melting temperature of aluminum alloys as compared to steel, often coexisting in a design, the outcome may be catastrophic for the former (Figure 32). For aluminum, however, much higher heat input is necessary to bring the same mass of metal to a given temperature, compared with steel. This is caused by high thermal conductivity of aluminum, being about four times that of steel and its specific heat twice that of steel, resulting in higher heat transfer away from the source.

### 8.1. Fire Initiation—Thermic Sparking

Aluminum is non-sparking in all environments if struck against aluminum, stainless steel, or any other material. The purpose in using non-sparking metals is to prevent ignition of combustible or explosive materials from an impact-generated spark. There is, however, one known exception: when unpainted or uncoated aluminum is struck by or strikes rusty ferrous metals, sparks may result [147]. Therefore, to avoid any possibility of sparking, where it is likely that aluminum may be struck by rusty ferrous metals, protective coatings such as paint are recommended.

Even if pure, non-ferrous aluminum is used, sparks can occur during an aluminothermic reaction, also called a thermic reaction. Such a reaction occurs when an aluminum particle and a metal oxide, such as rust, are ignited by a heat source and chemically burn as a “Class D” fire (i.e., combustible metal).

### 8.2. Exposure to Temperatures Exceeding the Melting Range

The solid bulk aluminum alloys exposed in air to temperatures exceeding the liquidus convert to molten state but are not subjected to burning, generating smoke or hazardous fumes. After fire is extinguished the metal remains as a re-solidified pool. Similarly, temperatures leading to semisolid state will result in an integrity lost by a design. The resistance of aluminum to burning is controlled by a number of national standards including ASTM. This is in contrast to fine powders or flakes of aluminum, which are highly flammable and oxidize exothermically. The ignition behavior of aluminum powder is similar to other finely divided materials including iron and titanium, which also readily oxidize exothermically while in the powder form.

### 8.3. Deterioration of Alloy Mechanical Properties during Fire

When structural collapse does not occur as a result of a fire, there is a need to evaluate the residual properties of overheated material to assess whether the structures should be dismantled, repaired, or directly reused. The influence of fire on mechanical properties depends on temperature and exposure time. The key difference from thermal stability issues discussed in this report is that alloys exposed to fire may not be designed at all for high temperature service. Therefore, even relatively low temperature such as 150 °C may lead to very poor performance during fire and the permanent property deterioration after fire.

There are a number of studies where the heat exposure on aluminum alloys that are not designed for high-temperature service, were evaluated. The objective was to accurately assess the post-fire performances of aluminum alloy structures. The models with predictive equations were developed, combining an influence of hardening factors and temperature on the material stress−strain relationship [148].

**Figure 33:**
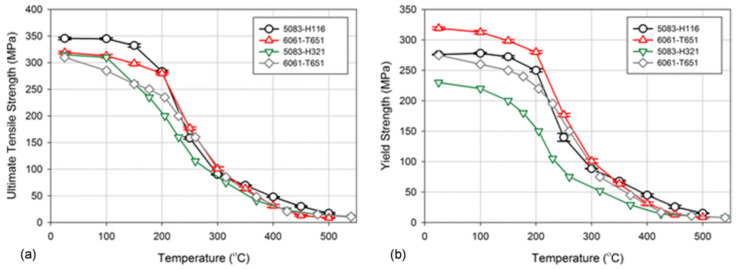
Degradation of tensile properties of 5083−H116 and 6061−T651 alloys during exposures to heat, expressed through ultimate tensile strength (**a**) and yield stress (**b**) [10].

An example of research where an existing constitutive model for creep was modified in order to be used for fire-exposed alloys involved AA5083−O/H111 and AA6060−T66 grades [149]. For temperature range of 170 to 380 °C the model predicted properties for the 5xxx series alloys. The same AA5083−H116 and AA6061−T651 marine-grade aluminum alloys were subject of extensive mechanical testing to determine the residual mechanical behavior after fire exposure [150] (Figure 33). The constitutive models were developed as a series of sub-models to predict (i) microstructural evolution, (ii) residual yield strength, and (iii) strain hardening after fire exposure. The properties of AA5000 series following a simulated fire exposure were evaluated in [151]. The 5xxx series alloys with different tempers resulted in residual strengths between 85 and 157 MPa following the fire exposure. Most alloys exhibited structural recovery between 100 to 280 °C followed by recrystallization between 300 to 340 °C. However, the AA5456−H116 alloy, which has the highest magnesium content, maintained 60% of room temperature yield strength. This alloy underwent recovery but did not have a clear recrystallization, preserving strength. Another study [152] found that the mechanical properties of AA6061−T6 were drastically reduced after exposure to temperatures exceeding 300 °C. For AA7075−T73, reduction in properties took place at lower temperature of 200 °C. An additional factor affecting post−fire mechanical properties of these two grades was a cooling rate from a relatively high fire temperature.

## 9. Concluding Remarks

Thermal stability is becoming the next frontier for aluminum alloys; understanding and overcoming limitations in this area would lead to substantial expansion in their structural applications, especially in aerospace and automotive sectors.

As practically all aluminum alloys are thermally unstable, with their properties being affected, to some extent, by service temperature and time, thermal stability is of concern throughout the entire temperature range of their applications. This includes grades essentially used at room temperatures, as is the case with aircraft components that may become warm due to exposure to sun, due to aerodynamic heating or heat transferred from engines, which can deteriorate alloys properties over years of service.

The major challenge in thermal stability of aluminum is, however, in increasing the upper service limit of high-temperature grades. There are efforts to develop new alloys with thermally stable microstructure through alloying aluminum with a variety of elements, in particular, transition, and rare earth metals. In parallel, there are efforts to generate more stable microstructure through novel processing routes, such as rapid solidification, powder metallurgy, mechanical alloying and additive manufacturing, engineering alloys in a liquid state prior to casting, and post-casting treatments.

The ultimate goal is to overcome the present knowledge and manufacturing barriers and develop aluminum alloys with superior properties that remain stable across the temperature and time space, required by modern designs.

## Appendix A. Diffusivities and Phase Diagram Data for Alloying Elements in Aluminum

Arrhenius equation for diffusion coefficient:

D = D_o_ exp(−Q)/RT), R = 8.314 Jmol^−1^K^−1^

D values at 500 °C were calculated in this work based on data cited in table

**Table A1:** Measured diffusivities of selected elements along with Al self-diffusion (data calculated are indicated).

Element	Group−Period	Pre−Exponential, D_o_	Activation Enthalpy, Q		D at 500 °C (773 K)	Temp. Range, °C	References
-	-	m^2^s^−1^	kJmol^−1^	eVatom^−1^	m^2^s^−1^	-	-
Al_self_	13−2	1.37 × 10^−5^	124	1.29	5.7 × 10^−14^	227−527	[153]
		1.71 × 10^−5^	142	1.47	4.34 × 10^−14^	450−650	[32]
		6.6 × 10^−6^	124	1.29	2.75 × 10^−14^	Calculated	[154]
Li	1−1	3.7^+1.30^_−0.28_× 10^−5^	126.1 ± 5.2	1.31 ± 0.05	1.11× 10^−13^	150−240	[155]
		4.5× 10^−4^	139.3	1.44	1.73× 10^−13^	310−597	[156]
Si	14−2	2.02^+0.97^_−0.66_ × 10^−4^	136 ± 3	1.41	1.3 × 10^−13^	480−620	[35]
		8.3 × 10^−7^	78.53	0.81	4.32 × 10^−13^	475−550	[157]
		1.19 × 10^−5^	122	1.27	6.75 × 10^−14^	Calculated	[158]
Mg	2−2	6.23 × 10^−5^	115 ± 1.15	1.19 ± 0.012	1.05 × 10^−13^	-	[159]
		1.0 × 10^−4^	131	1.35 ± 0.05	1.40 × 10^−13^	-	[160]
		1.49 × 10^−5^	121	1.25	9.92 × 10^−14^	-	[161]
		6.6 × 10^−5^	124 ± 1.45	1.29 ± 0.015	2.75 × 10^−13^	-	[162]

**Table A2:** Measured diffusivities for transition elements in aluminum (data calculated are indicated).

Element	Group−Period	Pre−Exponential D_o_	Activation Enthalpy, Q		D at 500 °C (773 K)	Temp Range, °C	Reference
-	-	m^2^s^−1^	kJmol^−1^	eVatom^−1^	m^2^s^−1^	-	-
Ti	4−3	1.12 × 10^−1^	261.5	2.71	2.38 × 10^−19^	-	[163]
		9 × 10^−5^	223.8	2.32	6.77 × 10^−20^	Calc	[36]
Zr	4−4	7.28^+10.4^_−4.29_ × 10^−2^	242	2.51	3.2 × 10^−18^	531−640	[164]
V	5−3	6.05 × 10^−12^	82.0	0.85	1.74 × 10^−17^	-	[165]
		1.6× 10^0^	303	3.14	5.35 × 10^−21^	-	[163]
		1 × 10^−4^	241	2.50	5.18 × 10^−21^	Calc	[36]
Nb	5−4	1.66 × 10^−11^	82.22	0.85	4.61 × 10^−17^	350−480	[166]
Cr	6−3	3.01 × 10^−11^	64.4	0.67	1.0 × 10^−15^	250−605	[167]
		10	282	2.92	8.78 × 10^−19^	-	[168]
		6.75 × 10^−1^	261.5	2.71	1.44 × 10^−18^	-	[161]
		7 × 10^−5^	199.7	2.07	2.24 × 10^−18^	Calc	[36]
Mo	6−4	1.4 × 10^−3^	250	2.59	1.79 × 10^−20^	-	[169]
Mn	7−3	1.04 × 10^−2^	211	2.19	5.73 × 10^−17^	460−660	[170]
		2.2 × 10^−5^	120.5	1.25	1.58 × 10^−13^	450−650	[32]
		1.35 × 10^−2^	211	2.19	7.44 × 10^−17^	-	[161]
		2 × 10^−5^	166.9	1.73	1.05 × 10^−16^	Calc	[36]
Fe	8−3	4.1 × 10^−13^	58.16	0.61	5.0 × 10^−17^	350−630	[171]
		7.7 × 10^−1^	221	2.29	8.95 × 10^−16^	-	[168]
		1.35 × 10^−2^	190	2.00	1.95 × 10^−15^	-	[172]
		3 × 10^−5^	145.7	1.51	4.28 × 10^−15^	Calc	[36]
Co	9−3	1.41 ± 1.37 × 10^−2^	168.8 ± 6.75	1.75 ± 0.07	5.52 × 10^−14^	-	[173]
		1.1 × 10^−10^	83.26	0.86	2.6 × 10^−16^	350−630	[171]
		1.93 × 10^−2^	168	1.75	8.57 × 10^−14^	-	[161,168]
		1.6 × 10^−6^	112.9	1.17	3.75 × 10^−14^	Calc	[36]
Ni	10−3	4.4 ± 3.l × 10^−4^	146 ± 5.8	1.51 ± 0.06	5.99 × 10^−14^	-	[173]
		2.9 × 10^−12^	65.69	0.68	1.0 × 10^−16^	350−630	[171]
		4.1× 10^−4^	144.7	1.50	6.83 × 10^−14^	-	[161]
		2 × 10^−4^	137.0	1.42	1.1 × 10^−13^	Calc	[36]
Cu	11−3	6.47 × 10^−5^	135.0 ± 1.12	1.40 ± 0.011	4.88 × 10^−14^	-	[174]
		4.37 × 10^−6^	120	1.25	3.39 × 10^−14^	-	[158]
		2.9 × 10^−5^	130 ± 6.7	1.35 ± 0.07	4.76 × 10^−14^	-	[175]
		4.44 × 10^−5^	113.8	1.18	9.06 × 10^−13^	Calc	[176]
Ag	11−4	1.18 × 10^−5^	116.4 ± 0.59	1.21 ± 0.006	1.61 × 10^−13^	-	[174]
Zn	12−3	2.59 × 10^−5^	120.7 ± 0.56	1.25 ± 0.006	1.80 × 10^−13^	-	[174]
		2.0 ± 0.8 × 10^−5^	120.7 ± 1.92	1.25 ± 0.02	1.39 × 10^−13^	-	[173]
		1.0 × 10^−4^	130	1.34	1.6 × 10^−13^	-	[177]

**Table A3:** Measured diffusivities for rare-earth elements in aluminum (data calculated are indicated).

Element	Group−Period	Pre−Exponential, D_o_	Activation Enthalpy, Q		D at 500 °C (773 K)	Temp Range, °C	Reference
-	-	m^2^s^−1^	kJmol^−1^	eVatom^−1^	m^2^s^−1^	-	-
Sc	3−3	1.90 × 10^−4^	164 ± 9	1.7 ± 0.09	1.57 × 10^−15^	300−400	[99]
		7.2 ± 6.0 × 10^−4^	176 ± 9	1.92 ± 0.09	9.20 × 10^−16^	400−450	[41]
		5.31 × 10^−4^	173	1.79	1.1 × 10^−15^	540−655	[178,179]
		2.34 ± 2.16 × 10^−4^	167 ± 6	1.73 ± 0.06	1.37 × 10^−15^	475,550,625	[180]
		2.65 ± 0.84 × 10^−4^ (*)	168 ± 2	1.74 ± 0.02	1.17 × 10^−15^	-	[180]
		5.0 × 10^−5^	165.9	1.72	3.08 × 10^−16^	Calculated	[36]
La	Lan	1.3 × 10^−10^	113.0	1.17	3.00 × 10^−18^	-	[39]
Ce	Lan	1.89 × 10^−10^	111.3	1.15	5.69 × 10^−18^	450−630	[39]
		4.0 × 10^−9^	130.2	1.35	6.3 × 10^−18^	Calculated	[181]
Pr	Lan	3.58 × 10^−11^	99.9	1.04	6.35 × 10^−18^	520−630	[39]
Nd	Lan	4.80 × 10^−11^	105.0	1.08	3.85 × 10^−18^	450−630	[39]
Sm	Lan	3.45 × 10^−11^	95.7	0.99	1.18 × 10^−17^	-	[39]

(*) results of combining measurements in [157,180] with literature data.

Additional diffusion data:

D_Y_ = 3.56 × 10^−17^ m^2^s^−1^ at 600 °C [182]

D_Er_ = (4 ± 2) × 10^−19^ m^2^ s^−1^ at 300 °C [100]

D_Yb_ = (6 ± 2) × 10^−17^ m^2^ s^−1^ at 300 °C [100]

**Table A4:** Phase diagram data for alloying elements with aluminum.

Element	Group-Period	Density	Melting Point	Al−X Phase Diagram ^(3)^	React Temp.	Eutectic Point	Solid Solubility	Solid Solubility	Phase Formed	Major Reference
-	-	g/cm^3^	°C	-	°C	wt.%	at.%	wt.%	-	-
Non−transition elements										
Li	1−1	0.534	180.5	Eut	596	7.51	14.5	4.18	Al_3_Li	[183]
Mg	2−2	1.74	650	Eut	450	19.51	18.9	9.55	Al_3_Mg_2_	[184]
Ca	2−3	1.55	842	Eut	616	7.68	0.05	0.07	Al_4_Ca	[185]
Sr	2−4	2.64	777	Eut	654	~1.5	<0.05	<0.16	Al_4_Sr	[186]
Si	14−2	2.33	1414.6	Eut	577	12.6	1.49	1.55	Si	[187]
Transition metals										
Ti	4−3	4.51	1670	Per	665.4	-	0.78	1.38	Al_3_Ti	[188]
Zr	4−4	6.52	1854	Per	660.8	-	0.08	0.28	Al_3_Zr	[189]
Hf	4−5	13.3	2233	Per	662.2	-	0.19	1.22	Al_3_Hf	[190]
V	5−3	6.00	1910	Per	664	-	0.33	0.63	Al_3_V	[191,192]
Nb	5−4	8.57	2477	Per	661.4	-	0.05	0.75 ^(1)^	Al_3_Nb	[193]
Ta	5−5	16.4	3017	Per	668.0	-	0.04	0.27	Al_3_Ta	[194]
Cr	6−3	7.15	1907	Per	661.5	-	0.37	0.72	Al_7_Cr	[195]
Mo	6−4	10.2	2622	Per	661	-	0.17	0.6 ^(1)^	Al_12_Mo	[193,196]
W	6−5	19.3	3414	Per	660.5 ^(2)^	-	0.18	1.2 ^(1)^	Al_12_W	[193]
Mn	7−3	7.3	1246	Eut	658	1.21	0.67	1.35	Al_6_Mn	[197,198]
Fe	8−3	7.87	1538	Eut	655	1.8	0.02	0.04	Al_3_Fe	[199,200]
Co	9−3	8.86	1495	Eut	657	0.98	0.01	0.02	Al_9_Co_2_	[201,202]
Ni	10−3	8.90	1455	Eut	639.9	6.1	0.11	0.13	Al_3_Ni	[203]
Cu	11−3	8.96	1084.6	Eut	548.2	32.7	2.33	5.67	Al_2_Cu	[204]
Ag	11−4	10.5	962	Eut	566	70.58	23.8	55.53	Al_2_Ag	[205]
Zn	12−3	7.14	419.5	Eut	277	77.7	67	83	Zn	[206,207]
Rare earth metals										
Sc	3−3	2.99	1541	Eut	660	0.47	0.23	0.38	Al_3_Sc	[208]
Y	3−4	4.47	1522	Eut	639	9.25	0.05	0.16	Al_3_Y	[209]
La	Lan	6.15	920	Eut	628	15.7	0.01	0.05	Al_11_La_3_	[210,211]
Ce	Lan	6.77	799	Eut	644.5	10.6	~0.01	~0.05	Al_11_Ce_3_	[124,212]
Nd	Lan	7.01	1024	Eut	641	~12	<0.2	0.04	Al_11_Nd_3_	[213,214]
Er	Lan	9.07	1529	Eut	655	~6	<0.05	<0.3	Al_3_Er	[100,200]
Yb	Lan	6.90	824	Eut	625	21.1	0.02	0.16	Al_3_Yb	[100]
Th	Lan	11.7	1750	Eut	630	20.44	0	0	Al_7_Th_2_	[215]

(1) supersaturated, cooling rate 10^3^−10^4^ deg/s; (2) aluminum melting point—660.452 °C; (3) Type of the binary Al-X phase diagram: Eut—eutectic reaction, Per—peritectic reaction.
